# A network perspective of engaging patients in specialist and chronic illness care: The 2014 International Health Policy Survey

**DOI:** 10.1371/journal.pone.0201355

**Published:** 2018-08-13

**Authors:** Yi-Sheng Chao, Marco Scutari, Tai-Shen Chen, Chao-Jung Wu, Madeleine Durand, Antoine Boivin, Hsing-Chien Wu, Wei-Chih Chen

**Affiliations:** 1 Centre de recherche du centre hospitalier de l’Université de Montréal (CRCHUM), Université de Montréal, Montréal, Québec, Canada; 2 Department of Statistics, University of Oxford, Oxford, United Kingdom; 3 Graduate School of Agricultural and Life Sciences, University of Tokyo, Tokyo, Japan; 4 Département d'informatique, Université du Québec à Montréal, Montréal, Québec, Canada; 5 Centre de recherche du Centre hospitalier de l’Université de Montréal (CRCHUM), Université de Montréal, Montréal, Québec, Canada; 6 Department of family and emergency medicine, Faculty of Medicine, University of Montreal, Montreal, Quebec, Canada; 7 Taipei Hospital, Ministry of Health and Welfare, New Taipei city, Taiwan; 8 Department of Chest Medicine, Taipei Veterans General Hospital, Taipei, Taiwan; 9 Faculty of Medicine, School of Medicine, National Yang-Ming University, Taipei, Taiwan; 10 Institute of Emergency and Critical Care Medicine, National Yang-Ming University, Taipei, Taiwan; La Trobe University, AUSTRALIA

## Abstract

**Background:**

Patient engagement helps to improve health outcomes and health care quality. However, the overall relationships among patient engagement measures and health outcomes remain unclear. This study aims to integrate expert knowledge and survey data for the identification of measures that have extensive associations with other variables and can be prioritized to engage patients.

**Methods:**

We used the 2014 International Health Policy Survey (IHPS), which provided information on elder adults in 11 countries with details in patient characteristics, healthcare experiences, and patient-physician communication. Patient engagement or support was measured with eight variables including patients’ treatment choices, involvement, and treatment priority setting. Three types of care were identified: primary, specialist and chronic illness care. Specialists were doctors specializing in one area of health care. Chronic illness included eight chronic conditions surveyed. Expert knowledge was used to assist variable selection. We used Bayesian network models consisting of nodes that represented variables of interest and arcs that represented their relationships.

**Results:**

Among 25,530 participants, the mean age was 68.51 years and 57.40% were females. The distributions of age, sex, education, and patient engagement were significantly different across countries. For chronic illness care, written plans provided by professionals were linked to treatment feasibility and helpfulness. Whether professionals contacted patients was associated with the availability of professionals they could reach for chronic illness care. For specialist care, if specialists provided treatment choices, patients were more likely to be involved and discuss about what mattered to them.

**Conclusion:**

The strategies to engage patients may depend on the types of care, specialist or chronic illness care. For the study on the observational IHPS data, network modeling is useful to integrate expert knowledge. We suggest considering other theory-based patient engagement in major surveys, as well as engaging patients in their healthcare by providing written plans and actively communicating with patients for chronic illnesses, and encouraging specialists to discuss and provide treatment options.

## Background

Engaging patients in treatment decisions, self-care, and other aspects of health care is useful to improve health outcomes and improve health care quality.[1–4] Patient engagement is “to promote and support active patient and public involvement in health and healthcare and to strengthen their influence on healthcare decisions, at both the individual and collective levels”.[5] Patients should be engaged in health care for ethical reasons.[6, 7] Patient engagement is also fundamental for patient safety and patient centered care.[6] However, there are several problems in formalizing the concept of patient engagement and its practice. Firstly, patient engagement is a term that not only involves many aspects of health care systems and patient-physician communication but also covers a broad spectrum of issues.[4, 6, 8] The precise measurement of patient engagement remains a work in progress for lacking uniform definitions[4], diverse measurement tools and distinct theoretical frameworks.[4, 9, 10] Taking the widely cited patient engagement framework by Carman et al. for example, the efforts of engagement can be described in two dimensions, the levels and the continuum of engagement.[4] In Carman et al.’s model, there are at least nine combinations of engagement levels and intensities.[4] A variety of activities can be included in Carman et al.’s model and a wide range of initiatives can be seen as engagement interventions.

Secondly, patient engagement is closely related to patients’ experiences in health care and sensitive to contextual factors, such as physician behaviour, patient characteristics and patient-physician communication.[11] The connections between patient engagement and the contexts, such as healthcare setting and illness, have been considered, but the effect sizes and relationships require further study.[4] In Canadian contexts, we recognize that patients set engagement priorities in the access to primary care, self-care support, patient participation in clinical decisions, and partnership with community among Canadian patients.[2] However, we are concerned that this result may not be applicable to patients in other countries or health systems. Thirdly, while prioritizing patient engagement is an important objective in a healthcare organization, it may conflict with other organizational initiatives and delay other patient-centred activities.[12] We think it important to understand the interactions between measures of engagement and other factors.[12]

Lastly, there are different and diverse measures of patient engagement proposed.[4, 6] Carman et al. proposed the measurement of individual, organizational and policy level engagement, such as whether patients received information, whether patients asked about their preferences, and treatment decisions.[4] Factors, such as patients’ motivation and knowledge, are considered to influence the level of patient engagement[4]. There are also a variety of other related factors discussed in the literature.[4, 6, 7] For example, the review by the World Health Organization lists important factors such as illness severity and tasks encountered by clinicians and patients.[6] The large numbers of patient engagement measures and related factors raise the concern that these measures or priorities may conflict with each other.[6]

In order to study to what extent patient engagement is sensitive to healthcare settings and whether different measures of patient engagement interact or are connected to each other, we think it necessary to pilot a study with available data. We aim to introduce a network approach by modeling patient engagement through Bayesian networks[13] with the 2014 Commonwealth Fund International Health Policy Survey (2014 IHPS) of Older Adults, which is one of the first international surveys that focus on patient engagement and other related measures at the same time.[14] The objectives of this study are to 1) describe overall relationships between patient engagement, individual characteristics and health systems across 11 countries in a network perspective; 2) identify potential measures to engage patients through intervening individual or health system factors; and 3) distinguish factors that are extensively linked to potential outcomes and may serve as engagement priorities that may lead to better health.

## Methods

### Data set

The IHPS has been a cross-sectional survey repeated annually by the Commonwealth Fund.[14] Questionnaires were administered to understand health related issues in different countries.[14] The questionnaires were revised from time to time.[14] Some variables were removed and new ones might be introduced. Specifically, the 2014 IHPS surveyed 25530 adults aged 55 years and over in 11 countries: Australia, Canada, France, Germany, the Netherlands, New Zealand, Norway, Sweden, Switzerland, the United Kingdom, and the United States.[14, 15] The samples were randomly selected in each country to obtain nationally representative statistics. Adults aged 55 years or over were called for telephone interview.[14] The measures in this survey included health status, healthcare use, healthcare costs and access, timeliness of care, care coordination and safety, doctor-patient relationship, health promotion, end-of-life planning, patient engagement, management of chronic conditions and caregiving.[14] The sampling frames, the rationales and theories of the included measures were reported by the Commonwealth Fund researchers.[14] The variables related to patient engagement and activation were first introduced to the IHPS in 2014 survey.[14] The theories for the patient engagement measures were discussed in the next section.

There were several important features in the 2014 IHPS data set. The first was that many questions were country-specific. This was because the health systems differed from country to country and several countries chose to add questions pertinent to their healthcare system.[15] For example, a section of the questionnaire was specifically used to ask the status of health insurance and insurance purchase. These questions were very useful to understand health insurance coverage in the United States, but not useful for other countries where healthcare insurance was administered by governments and provided to almost all citizens, such as the United Kingdom and Canada. For the levels of education, the titles of the secondary school diploma differed and the methods to report the types of degrees varied. The use of country-specific questions led to high proportion of ineligibility in some of the variables (see S1 Appendix for the proportions of missing data).

Also there was variability of exposure to healthcare systems among interviewees. Interviewees’ experiences in health care were asked in questions about primary care, specialist care, chronic illness care, hospitalization, emergency room use and end-of-life planning. Interviewees were mostly eligible to questions about primary care and the response rates were higher than those about hospitalization and emergency room use (see S1 Appendix for details). This also contributed to high proportions of ineligibility in some variables.

In addition to ineligibility to certain questions, this survey also had other limitations.[14] First, the response rates varied significantly according to countries and sampling frames (random-digit dialling or population registry) and the direction of bias was unknown.[14] The other limitation was that institutionalized adults were not sampled in the survey and so countries with higher rates of institutionalization might seem healthier than those with lower rates.[14]

### The measures of patient engagement or support in the 2014 IHPS

**Fig 1:**
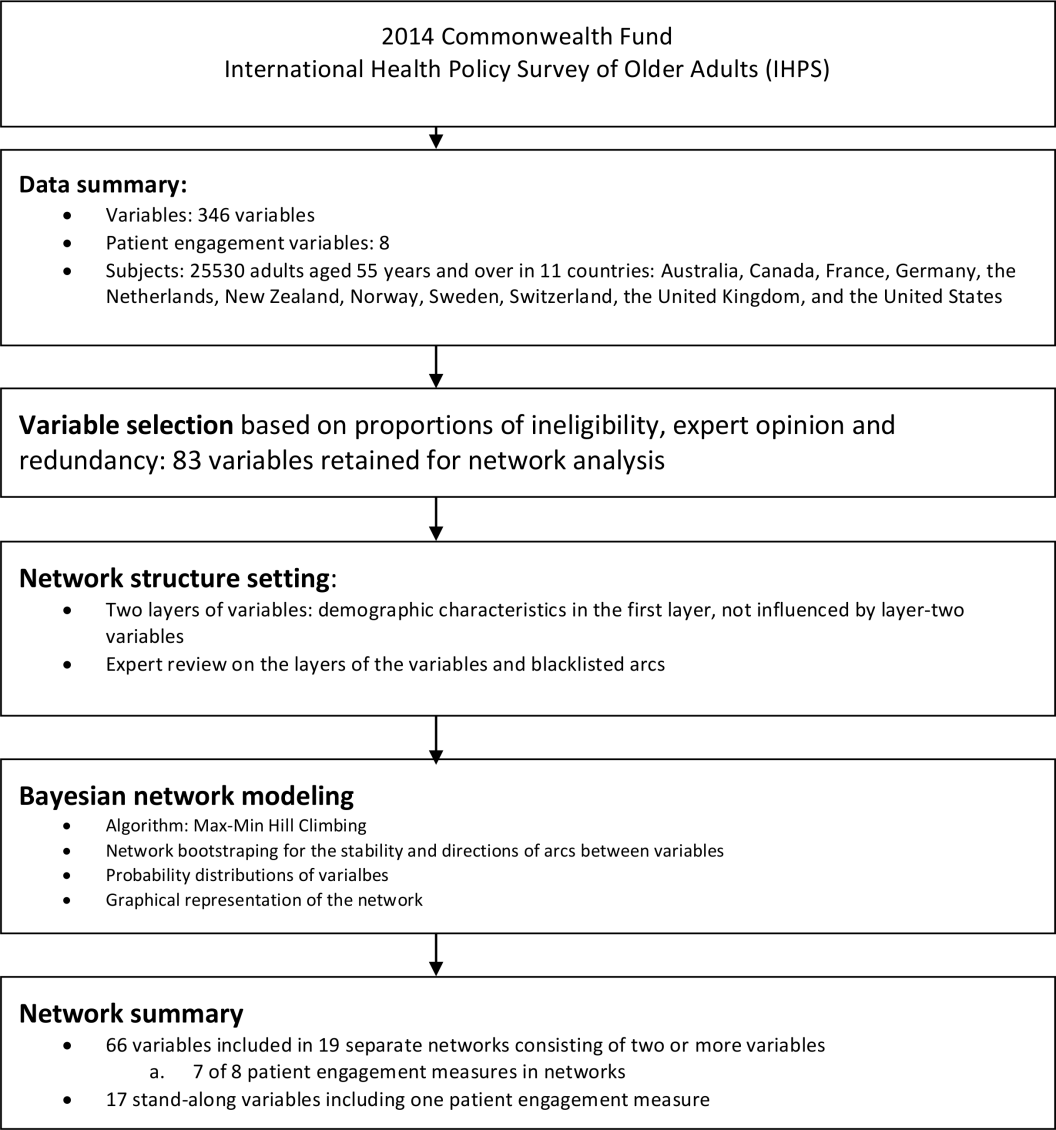
Flow chart of variable selection and network modeling with the 2014 Commonwealth Fund International Health Policy Survey of Older Adults.

**Fig 2:**
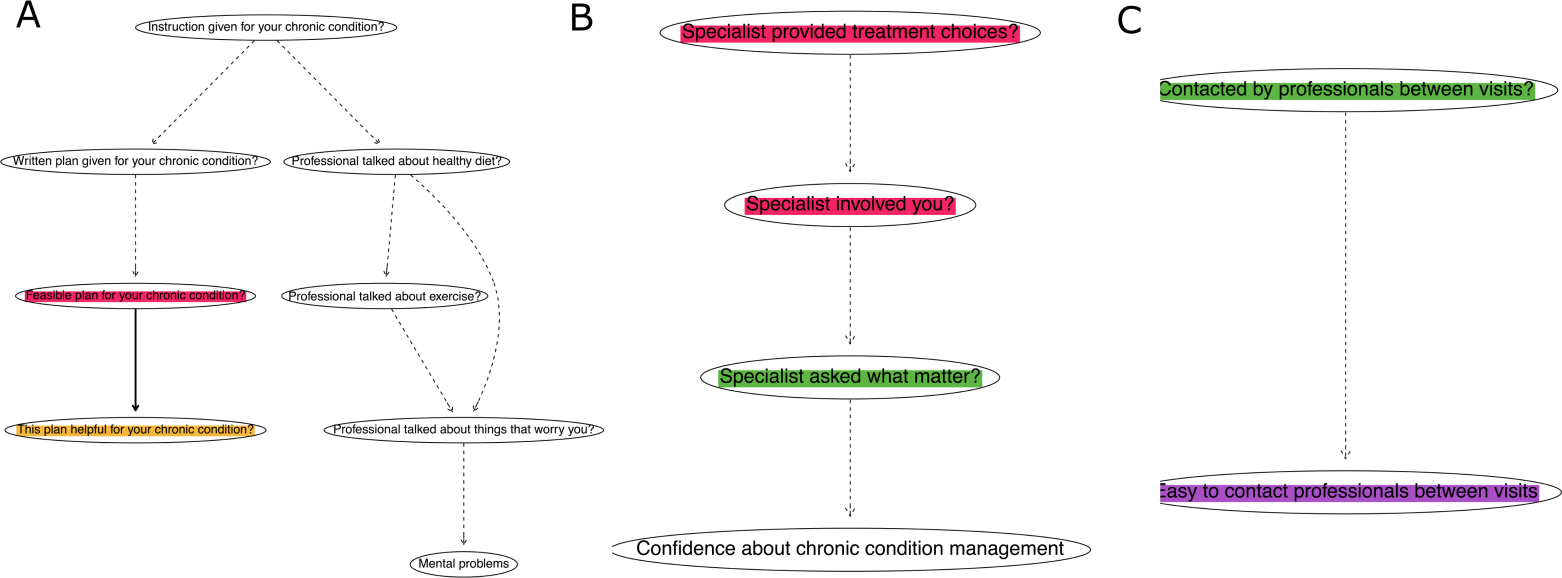
Networks of patient engagement, countries, and experience in health care. (a) The largest network consisting of eight variables, out of 83 variables. Note: The thicknesses of arcs are proportional to the absolute values of Spearman’s correlation coefficients between variables. See variable definitions in Table 1. (b) The network of the measures of patient engagement or support in specialist care. (c) The network of patient engagement in chronic illness care. Note: See Table 1 for variable definitions.

Patient engagement or patient support was measured by eight variables based on two theories, four related to chronic conditions and four related to specialist care in the 2014 IHPS.[14] At the time of variable creation, two important patient engagement theories were determined fit for the survey and selected by the Commonwealth Fund researchers, Patient Activation Measure and a team-based comprehensive model.[14] According to the Patient Activation Measure, a validated tool, patient engagement was related to patients’ knowledge, skills, beliefs, leadership and autonomy about the health care and treatment.[16, 17] In the team-based comprehensive care model, the continuity of care, communication with patients, patients’ involvement with providers, and access to physicians, were important factors.[18] Based on these two patient engagement or activation theories, the exact variables used in the 2014 IHPS included 1) how often specialists provided patients with treatment choices (variable short name: SP_Rx_choice); 2) how often specialists involved patients as much as patients wanted (SP_Involvement); 3) how often specialists asked what mattered to patients (SP_What_matters, only in the Netherlands); 4) how often specialists gave patients’ family and relatives the opportunity to involve in care as much as patients wanted (SP_Family_involved only in Sweden); 5) whether patients had treatment plans that they could carry out in their daily life (CC_Feasible_plan); 6) whether the treatment plans helped control or manage conditions (CC_Helpful_plan); 7) whether there were health care professionals who contacted patients to see how things were going between doctor visits (CC_contacted); and 8) whether there were healthcare professionals that patients could easily contact to ask questions or get advice between doctor visits (CC_DR_for_question) in Table 1.[14] To link these measures to existing patient engagement literature, we classified the variables into three core components of patient participation: shared decision-making, self-care and autonomy, and patient having critical self-knowledge based on the Patient Activation Measure and the team-based comprehensive care model[9, 16–18] in Table 1. The variables of different patient engagement categories were designated with different colors in Table 1 and in Figs 1 and 2.

**Table 1:** The measures of patient engagement or support and related variables in the Commonwealth 2014 International Health Policy Survey.

**Short description in Fig 2 and S2 Appendix**	**Variables in S3 Appendix**	**Patient engagement classification**	**Color labels for patient engagement measures**	**Short names used S4 Appendix**	**Proportions of ineligibility**	**Proportions of missing data**	**Layers**	**Original questions**	**Responses**
**Easy to contact professionals between visits**	q1425a2	Mixed	Purple	CC_DR_for_question	28.4%	2.1%	2	Q1425_A2. Between doctor visits, is there a health care professional You can easily contact to ask a question or get advice about your health condition(s)?	Yes; No
**Contacted by professionals between visits?**	q1425a1	Patient as having critical self-knowledge	Green	CC_contacted	28.4%	0.7%	2	Q1425_A1. Between doctor visits, is there a health care professional Who contacts you to see how things are going?	Yes; No
**Specialist asked what matter?**	q1065a3	Patient as having critical self-knowledge	Green	SP_What_matters	97.5%	0.1%	2	Q1065A3. When you have received care or treatment from specialists, how often did they Ask what matters to you. Would you say always, often, sometimes, rarely or never?	Always; Often; Sometimes; Rarely or never; Not applicable
**This plan helpful for your chronic condition?**	q1423	Self care and autonomy	Orange	CC_Helpful_plan	57.4%	1.7%	2	Q1423. Has this plan helped you control or manage your _____? Would you say …?	Not at all; Only a little; Some; A lot
**Specialist involved your family?**	q1065a4	Self care and autonomy	Orange	SP_Family_involved	85.5%	0.5%	2	Q1065A4. When you have received care or treatment from specialists, how often did they give your family and relatives the opportunity to be involved in your care as much as you wanted. Would you say always, often, sometimes, rarely or never?	Always; Often; Sometimes; Rarely or never
**Feasible plan for your chronic condition?**	q1422	Shared decision-making	Red	CC_Feasible_plan	28.4%	1.2%	2	Q1422. Do you have a treatment plan for your _____ that you can carry out in your daily life?	Yes; No
**Specialist involved you?**	q1065a2	Shared decision-making	Red	SP_Involvement	42.6%	1.4%	2	Q1065A2. When you have received care or treatment from specialists, how often did they Involve you as much as you want to be in decisions about your treatment or care. Would you say always, often, sometimes, rarely or never?	Always; Often; Sometimes; Rarely or never;Not applicable
**Specialist provided treatment choices?**	q1065a1	Shared decision-making	Red	SP_Rx_choice	42.6%	1.2%	2	Q1065A1. When you have received care or treatment from specialists, how often did they Tell you about treatment choices. Would you say always, often, sometimes, rarely or never?	Always; Often; Sometimes; Rarely or never; Not applicable
**Instruction given for your chronic condition?**	q1420a3			CC_Instruction	28.4%	1.3%	2	Q1420A3. During the past year, when you received care, has any health care professional you see for your _____—Given you clear instructions about symptoms to watch for and when to seek further care or treatment?	Yes; No
**Written plan given for your chronic condition?**	q1420a4			CC_Written_plan	28.4%	1.3%	2	Q1420A4. During the past year, when you received care, has any health care professional you see for your _____—Given you a written plan to help you manage your own care? (IF NEEDED: By managing your own care, we mean knowing what …	Yes; No
**Age**	q715			Age	0%	0%	1	Q715. [P.N.—HIDDEN COMPUTE FOR AGE.]	
**Sex**	q725			Sex	0%	0%	1	Q725. RESPONDENT SEX	Male; female
**College education**	college			College					
**Community types**	q615				57.1%	0%	1	Q615. COMMUNITY TYPE	
**Regions in countries**	q630				0%	0%	1	Q630. REGION	
**Linguistic regions**	q640				0%	0%	1	Q640. LINGUISTIC REGIONS	
**Swiss languages**	q726				92.9%	0%	1	Q726. RESPONDENT LANGUAGE	
**How confident about chronic condition management?**	q1424			CC_confidence	97.2%	0.3%	2	Q1424. How confident are you that you can control and manage your health problems?	Very confident; Confident; Not very confident; Not at all confident
**Professional talked about exercise?**	q1480a2			Exercise	0%	0.5%	2	Q1480A2. During the past 2 years, has any health professional talked with you about …?—Exercise or physical activity	Yes; No; Have not seen a doctor in past 2 years
**Professional talked about healthy diet?**	q1480a1			Healthy_diet	0%	0.5%	2	Q1480A1. During the past 2 years, has any health professional talked with you about …?—A healthy diet and healthy eating	Yes; No; Have not seen a doctor in past 2 years
**Mental problems**	q1415a5			Mental	0%	0.5%	2	Q1415_A5. Have you ever been told by a doctor that you have Depression, anxiety or other mental health problems?	Yes; No
**Professional talked about things that worry you?**	q1480a3			Stress_sources	0%	0.7%	2	Q1480A3. During the past 2 years, has any health professional talked with you about …?—Things in your life that worry you or cause stress	Yes; No; Have not seen a doctor in past 2 years
**Country**	q600			Country	0%	0%	1	Q600. COUNTRY CODE	Australia; Canada; France; Germany; Netherlands; New Zealand; Norway; Sweden; Switzerland; United Kingdom; United States
**Because of cost, dentists not visited**	q810a4			Cost_no_dentist	79.4%	0.04%	2	Q810A4. During the past 12 months, was there a time when you Did not visit a dentist when you needed to because of the cost?	Yes; No
**Regular Dr?**	q915			Regular_Dr	0%	0%	2	Q915. REGULAR DOCTOR OR PLACE	HAS REGULAR DOCTOR/GP/NP, PA; HAS REGULAR PLACE; NO REGULAR DOC/PLACE
**Stroke**	q1415a9			Stroke	94.1%	0%	2	Q1415_A9. Have you ever been told by a doctor that you have Stroke?	Yes; No
**Heart disease**	q1415a2			Heart disease	0%	0.6%	2	Q1415_A2. Have you ever been told by a doctor that you have Heart disease, including heart attack?	Yes; no
**Diabetes**	q1415a3			Diabetes	0%	0.5%	2	Q1415_A3. Have you ever been told by a doctor that you have Diabetes?	Yes; no

Note: q1065a3 and q1065a4 applicable only to interviewees in the Netherlands and Sweden respectively. Colors corresponding to those in Fig 2.

#### Data cleaning and variable definitions

If subjects were not eligible for specific questions, their replies were coded as ineligible (see S1 Appendix for proportions of ineligibility of the variable). For example, questions specifically designed for Swiss interviewees were not applicable for individuals from other countries. Those who had not experienced hospitalization were documented as ineligible for questions about hospital admissions. The following categories of answers to all questions were recoded as missing: “don’t know”, “not sure”, “refused”, or “decline to answer” (see S1 Appendix for proportions of missing information in each variable). This data cleaning principle was consistent with that taken in the article by the Commonwealth Fund researchers.[14]

Education attainment was recoded across countries to see whether the respondents obtained at least some form of college or post-secondary education (see S1 Appendix for the list of 185 variables, questions and network numbers). Primary care was patients’ usual source of care provided by whether general practitioners, doctors, nurse practitioners, physician assistant, or others. Specialists were defined as doctors that specialized in one area of health care such as surgery, heart, allergy or mental health (only in Australia, Canada, France, Germany, New Zealand, Norway, Sweden, Switzerland, the United Kingdom, and the United States) or neurology (only in the Netherlands).[15] Health conditions that were included in the questionnaire included diabetes, high blood pressure, heart disease, chronic lung disease, depression, anxiety, other mental health problems, cancer, and joint pain or arthritis.[15] General health status was rated as excellent, very good, good, fair, and poor.[15]

### Bayesian network modeling

To understand the overall relationships between the variables in the 2014 IHPS, we used a probabilistic graphical model, known as Bayesian networks for the analysis.[13, 19, 20] Bayesian network analysis adopted Bayesian statistics for network analysis.[19] In Bayesian networks, variables were presented as nodes and the arcs were used to present the relationships between variables in terms of conditional probabilities.[13, 19, 20] Bayesian statistics built on Bayes’ theorem that described the relationships between prior probabilities and posterior probabilities based on given events were used to propagate information between nodes or variables.[19] In other words, the probability distribution of one variable was related to its parent variable. If two variables were related only because they had the same parent variable, they were interpreted as conditionally independent in Bayesian network modeling.[13, 19, 21] Bayesian network modeling could be applied to identify the parent variables and the strengths of the linkages to parent variables in terms of conditional probabilities.[13, 19, 21]

In general, the advantages of using Bayesian network include the ability of handling incomplete data, structural learning, integrating information from diverse sources, presenting variable relationships with visual clues, and fast responding to post-analysis queries.[22] Sometimes causal interpretation can be inferred from the arcs in the networks.[20] The Bayesian network had been used in many disciplines, ranging from health systems research,[21] medicine,[23, 24] biology,[25] and sociology[26] to study the conditional dependencies between variables. The modeling process was shown in Fig 1.

Specific to the research on patient engagement measures and their interactions with contextual factors, the advantage of using Bayesian network modeling included the potential to discover latent structures of the relationships between variables.[13, 19, 20] The identified latent structure could be visualised in networks.[13, 19, 20] In contrast, regression models required researchers to treat one single variable as outcome and others as predictors.[27, 28] For regression models, it was assumed that the effects of the predictors were estimated simultaneously to one single outcome. This led to difficulties in understanding the interactions between patient engagement measures and contextual factors with conventional regression models. The assumptions of regression models might not be applicable to patient engagement variables. We considered the application of Bayesian network modeling important for the exploration of the interactions between patient engagement measures and other contextual factors.

### Model development process and expert opinion

The Bayesian network models were built and reviewed in a way similar to the development process previously proposed.[21, 29] After data cleaning and missing data assessment,[29] the redundant variables or administrative variables were searched and not included for analysis (see Reasons for exclusion column in S1 Appendix). The variables that were derived by the Commonwealth Fund and were not labeled with clear definitions or not matched to the questions in the questionnaire were excluded from analysis. The Bayesian network model implementation was supervised by an expert panel based on the following steps.[30] The experts included a patient engagement expert (AB), an epidemiologist (MD), a data analyst (YSC), and a Bayesian network specialist (MS), who created the *bnlearn* package for the implementation of Bayesian network analysis within R environment.[13, 20] Three of them were also medical doctors with experiences in medical practices (AB, MD, and YSC). MD and MS were invited by AB and YSC for their expertise in Bayesian network modeling and epidemiological investigation respectively. The first step was that all variables were used for data-driven model building and assessed for adequacy (see S2 Appendix for original model output). Second, the expert-driven perspectives were introduced to 1) select the necessary variables for further model selection, and 2) identify essential links between variables. After discussion and model re-specification, the conclusion by the expert panel was as follows. The country-specific variables were determined inadequate and not used for the final model. Because the amount of health care spending could not be harmonized across countries, this variable was not selected for analysis. The arcs to demographic characteristics and national health care systems were blacklisted (see the next paragraph about layering or blacklisting). There were no arcs deemed obligatory or whitelisted.[13] In other words, the expert opinions helped to identify ineligible variables only and did not impose assumptions about the network structure (see S1 Appendix for the variables included for the final model and reasons for exclusion).

### Layers of variables

There were restrictions on the directions between variables. First, the variables other than age (variable names available in S1 Appendix: q715), sex (q725), some college education (college), countries (q600), community types (q615), regions in countries (q630), linguistic regions (q640), and Swiss languages (q726), were not allowed to be directed to the above-mentioned variables. In other words, the other variables could not influence the probability distributions of above-mentioned demographic characteristics or health systems (layer-one variables in S1 Appendix). This technique was called blacklisting[13] or layering[24] to ensure there were no arcs directed from other variables to socio-demographic characteristics.

#### Bayesian network implementation

We constructed the Bayesian networks from the data set by the following steps: 1) apply one of the best heuristic algorithms, Max-Min Hill Climbing, for structural learning,[24] 2) test the stabilities and strengths of arcs in the networks using the average of 200 bootstrapped networks, 3) obtain the directions of arcs between variables from the averaged network, and 4) estimate the probability distributions in the created network and 4) illustrate the final networks.[13] To test the arc strengths and stabilities, there were 200 networks created based on the new data sets bootstrapped from original data.[13] The interviewees were resampled and the sample sizes of the bootstrapped data sets were the same as the original data set. For each bootstrapped data set, the structure of the graphical network was learned.[13] The strengths of the arcs were estimated by averaging the probability of the arcs presenting in the bootstrapped networks.[13]

### Correlations between variables and cross-group comparisons

In addition to Bayesian network modeling, the relationships between pairs of variables were also quantified by the correlation coefficients from Spearman’s correlation tests. The differences in continuous and categorical variables across countries or parent variables were also tested with one-way analysis of variance and Chi-square test, respectively. The p-values less than 0.05, two-tailed, were considered statistically significant. This study adopted R[31] (v. 3.10) and RStudio[32] for data analysis.

### Ethics review

This secondary data analysis was approved by the ethics review committee at the Centre Hospitalier de l’Université de Montréal.

## Results

Among all participants, the mean age was 68 years and 57.4% were females. The distributions of age in years, sex, college education, heart disease, diabetes, and the eight patient engagement measures were significantly different across 11 countries, as described in Table 2 (p<0.01 for all).

**Table 2:** The demographic characteristics of the subjects in the Commonwealth Fund 2014 International Health Policy Survey of Older Adults.

		Australia	Canada	France	Germany	Netherlands	New Zealand	Norway	Sweden	Switzerland	United Kingdom	United States	All
**N**		3310	5269	1500	928	1000	750	1000	7206	1812	1000	1755	25530
**Female (%)**		51.2%	63.4%	52.1%	61.2%	53.8%	53.7%	53.1%	59.0%	53.8%	50.7%	60.7%	57.4%
**Some college education (%)**		56.4%	53.0%	49.1%	45.2%	25.2%	53.5%	51.3%	40.6%	28.5%	35.4%	63.2%	46.5%
**Heart disease, including heart attack (%)**		13.1%	13.8%	10.7%	21.0%	19.3%	12.8%	16.6%	15.6%	14.3%	8.9%	19.5%	14.8%
**Diabetes (%)**		12.8%	17.1%	12.2%	14.4%	15.5%	11.3%	8.1%	11.0%	10.5%	12.3%	22.9%	13.6%
**Specialist provided treatment choices? (%)**	Always	36.1%	31.4%	10.9%	43.6%	42.6%	30.7%	18.6%	21.4%	38.7%	25.1%	45.5%	29.6%
	Often	13.3%	9.9%	28.8%	18.1%	9.9%	6.4%	10.0%	9.3%	8.0%	11.0%	11.5%	11.5%
	Sometimes	4.5%	5.6%	9.6%	7.3%	3.3%	4.5%	5.4%	4.9%	3.3%	4.0%	5.1%	5.2%
	Rarely or never	2.0%	5.1%	5.5%	10.1%	3.8%	2.4%	13.9%	7.6%	6.2%	2.3%	3.4%	5.7%
	Never see other doctors/place or needed coordination	1.9%	3.6%	0.5%	5.2%	5.0%	2.5%	9.0%	6.0%	8.8%	0.7%	1.9%	4.3%
	Not applicable	41.5%	43.6%	44.7%	13.6%	34.8%	52.4%	40.8%	48.8%	33.9%	56.7%	31.7%	42.6%
**Specialist involved you? (%)**	Always	38.3%	35.0%	10.7%	38.3%	39.9%	32.9%	27.1%	25.4%	43.5%	27.5%	49.2%	32.5%
	Often	12.7%	10.1%	32.0%	16.9%	8.0%	8.0%	14.1%	9.9%	7.5%	9.8%	10.1%	11.7%
	Sometimes	4.5%	5.4%	7.5%	8.8%	4.3%	4.3%	5.7%	4.0%	2.6%	4.1%	5.2%	4.8%
	Rarely or never	1.5%	3.5%	4.5%	11.0%	5.5%	0.8%	5.5%	5.2%	4.5%	1.3%	2.4%	4.1%
	Not applicable	1.0%	1.8%	0.4%	8.3%	6.7%	0.9%	3.5%	4.0%	6.6%	0.5%	0.6%	2.9%
**Feasible plan for your chronic condition? (%)**	Yes	49.6%	59.8%	42.9%	23.8%	30.3%	38.4%	32.6%	28.0%	35.5%	41.2%	69.4%	42.6%
**This plan helpful for your chronic condition? (%)**	Not at all	0.5%	0.6%	0.7%	1.2%	1.1%	0.3%	1.8%	0.8%	0.6%	0.6%	0.6%	0.7%
	Only a little	3.7%	3.0%	2.2%	0.9%	4.0%	1.7%	2.2%	2.3%	2.0%	3.6%	3.0%	2.7%
	Some	13.4%	11.5%	13.3%	8.1%	4.2%	9.5%	9.2%	8.5%	6.3%	12.3%	13.1%	10.2%
	A lot	30.2%	43.2%	26.5%	12.7%	19.3%	25.7%	17.6%	14.0%	26.0%	23.4%	51.1%	27.3%
**Contacted by professionals between visits? (%)**	Yes	16.3%	10.5%	15.7%	9.9%	15.8%	17.2%	8.0%	11.8%	6.6%	27.1%	24.8%	13.6%
**Easy to contact professionals between visits (%)**	Yes	47.3%	50.8%	36.3%	31.9%	56.2%	43.7%	35.6%	50.2%	42.8%	40.8%	69.3%	48.4%

Note: p< 0.01 for all variables, significantly different across countries. Categorical and continuous variables were compared with Chi-square and t tests respectively. Only selected categories were shown for limited spaces. The statistics were available in Osborn et al. (2014).

### Networks of patient engagement and other variables

Based on Bayesian networks modeling, networks were drawn to describe the connectedness between variables. For the 2014 IHPS data, it was possible to identify networks that could be generalized to the data from 11 countries. Not all variables were connected in one single network. There were 19 separate networks identified and 66 variables were included in these networks. The 66 variables were related to countries of residence, sex, age, health status, healthcare utilization, patient engagement, health behavior, and experiences in primary, specialist and chronic illness care (see S1 Appendix for details). Ten networks consisted of two or three variables. Eight networks consisted of four variables. The largest network consisted of eight variables, two of which were patient engagement variables about chronic illness care in Fig 2A. The patient engagement variables were included in three networks, except for the variable, “whether specialists involved patients’ family or relatives in their care” that did not connect to other variables.

### Networks of patient engagement by specialist and chronic illness care

The three networks consisted of any patient engagement variables were shown in Fig 2. There were two characteristics about the patient engagement variables. First, the patient engagement variables about specialist care tended not to connect with the engagement variables about chronic illness care or primary care (see S2 to S4 Appendices for all networks). The other was that patient engagement variables were only connected to two variables that were unrelated to patient engagement. The first variable connecting to patient engagement variables was “whether professionals provided written plans for your chronic condition” (not a patient engagement variable), which was connected to “feasible plan for your chronic condition” in Fig 2A. This connection was found in the largest network with eight variables. The other was “how confident about chronic condition management” (not a patient engagement variable), which was lined to a patient engagement variable, “how often specialists asked what mattered” in Fig 2B.

### Contexts and potential measures for intervention

#### Patient engagement and support in chronic illness care

The four measures of patient engagement or support in chronic illness care were interconnected with each other in two networks in Fig 2A and 2C. In chronic illness care, “whether professionals gave patients clear instructions about symptoms to watch for and when to seek further care” was linked to two variables, “whether they gave patients written plans to manage conditions” and “whether they talked about healthy diet and eating” (not a patient engagement variable) in Fig 2A.

Then “whether written plans were given for your chronic condition” was associated with “feasible plan for your chronic condition” in Fig 2A. This was to say that the patients with written plans were more likely to have feasible plans for their conditions than those without, with probabilities 82.7% and 47.9% respectively (S5 Appendix). The patients with feasible plans were more likely to rate them a lot helpful or somewhat helpful. In Fig 2C, if there were health professionals to contact patients to see how things were going, it was more likely to have professionals that patients could easily contact to ask a question or get advice for conditions in chronic illness care (see S3 Appendix for detailed statistics).

#### Patient engagement in specialist care

For patient engagement in specialist care, “how often specialists provided treatment choices” was directly linked to “how often specialists involved patients as much as patients wanted”. In Fig 2B, the more often specialists told patients treatment choices, the more likely patients were involved as much as patients wanted. Subsequently, the more often specialists involved patients as much as they wanted, the more often specialists asked what mattered to them. The frequency of specialists asking what mattered to patients linked to patients’ confidence in the control and management of health problems (not a patient engagement variable). In addition, how often specialists gave family or relatives the opportunity to be involved in patients’ care was asked only in Sweden and not linked to any other variables (see S6 Appendix for detailed statistics).

## Discussions

We identify networks of variables from a data set collected in 11 countries. Although not all eligible variables have been linked in a single network, seven of eight patient engagement variables are connected in three separate networks. We find the patient engagement variables about specialist care are not linked to the engagement variables about chronic illness care. The characteristics of individuals and primary care setting have limited influence on patient engagement. There are variables linked to multiple variables and may have the potential to be a target for intervention. For example, the parent variables linked to patient engagement and health outcomes in Fig 2.

The engagement theories adopted by the IHPS are the Patient Activation model[17] and a team-based practice model.[18] This may be the reason why the patient engagement questions are mostly related to shared decision-making, self-care and autonomy, and patient’s knowledge.[14] If categorizing according to Carman et al.’s model, this is direct-care level engagement limited to consultation and individual involvement.[4] The questions for the other two intensities of engagement, partnership and shared leadership in direct care, need to be developed and introduced, while organizational and policy-level measurement of patient engagement is lacking in the 2014 IHPS.

The results are important for several reasons. Firstly, this study is the first to show how the measures of patient engagement can be linked in networks based on the 2014 IHPS data collected in 11 healthcare systems. Measures of patient engagement are known for associations between each other[33] and extensive linkage to other contextual and individual factors.[6] In conventional regression models, one-to-one or multiple-to-one relationships are assumed between independent variables and an outcome.[13, 27, 28] Without adopting the assumptions of regression models, we used Bayesian network modeling to construct networks and demonstrate how patient engagement measures and health outcomes were interconnected in Fig 2. Among various contextual factors recognized for their importance in patient engagement,[34–36] our empirical research shows that two types of healthcare, specialist or chronic illness care, are important contextual factors that relate to the separation of networks.

Secondly, patient engagement activities about chronic illness care may have little connections with the engagement variables about specialist care. The contexts of health care seem to matter for patient engagement, although some researchers may take patient engagement as an inseparable idea that should be discussed in the same manner in all settings.[14] The recognition of different settings or contexts can help us to identify important and influential factors and prioritize engagement initiatives accordingly. The separation of engagement networks into different settings not only helps to better understand the role of patient engagement in health care, but also shows how to link the theories of patient engagement[4, 7] with real-world settings. This finding is supplemental to current knowledge in the factors that may influence patient engagement in the primary care.[6, 7] In the literature review by the World Health Organization, health literacy, physician attitudes, and health setting in terms of primary or secondary care are suggested to influence the level of patient engagement.[6] Our finding suggests that specialist or chronic illness care may be important targets for intervention in addition to primary or secondary care distinction.[6] In fact, some of the theories neglect the importance of care setting,[4] while our results show distinct networks for specialist and chronic illness care.

Thirdly, health status is not directly linked to patient engagement in specialist or chronic illness care. This does not confirm the evidence that directly links patient engagement to health status or outcomes.[37] We think there are several reasons for the discrepancies. Existing evidence may have limitations in inferring causation from engagement to better health because of confounding. Currently there are few randomized controlled trials on patient engagement regarding health outcomes.[2] Many of the effectiveness studies are observational.[7] Our finding suggests that it is possible to make faulty causal inferences if factors that lead to both improved health outcomes and better patient engagement are not adjusted for. For example, “giving instructions in chronic illness care” is leading a network in Fig 2A. As a parent variable, this variable is associated with two patient engagement measures, physical activities, and mental health, while these two patient engagement measures are conditionally independent of physical activities and mental health in a network perspective. If “giving instructions in chronic illness care” was not accounted for, it is possible to falsely identify a causal relationship between patient engagement and mental health. In addition to confounding, there are other unmeasured variables. Many aspects of physical or mental health that were not measured in the 2014 IHPS. For example, obesity was not measured as an outcome and mortality could not be studied in this survey. Other health outcomes or pathways that influence patient engagement and health status should be investigated in the future.

Lastly, there are no direct links between socioeconomic status and patient engagement or support. This contrasts previous findings on the significant associations between income and patient activation[17] or overall socioeconomic status and engagement.[38] Socioeconomic characteristics are also important for factors that may influence the levels of patient engagement, especially health literacy and illness severity.[6] We think our finding shows the differences between a network model and conventional regression-based approaches. The network model we adopt aims to construct a network of variables by integrating expert knowledge with empirical evidence and assessing conditional probabilities between all variables. To set up the model, we only reject implausible relationships, such as healthcare experience leading to demographic characteristics, and do not assume predictor-outcome relationships as researchers do with regression analysis. A network perspective is useful while various researchers are taking opposite views on patient engagement, whether to be a means to better outcomes[3, 37] or an outcome of system performance.[39] The results are particularly meaningful for researchers who would like to search or explore the role of patient engagement in a systematic manner, whether as a means or an end. Although the exact reason for the lack of associations between socioeconomic status and engagement is unclear, we think the application of network models to other data sets that have been examined with regression models will help to understand the role of socioeconomic status on both health outcomes and patient engagement.

This is also a first step in showing how engagement theories can be demonstrated in a network illustration that consists of patient engagement measures, contextual factors and individual characteristics. It is surprising to find that three core components of patient participation[9] may be sensitive to the contexts and do not necessarily reinforce each other. For example, the treatment choices provided by specialists are associated with the involvement in patients’ own care, but not linked to patients’ autonomy in chronic illness care. The role of healthcare contexts and how they mediate the effects of patient engagement need to be further investigated.

### Implications

There are several research implications. Firstly, the measures of patient engagement or support do not necessarily interact with each other. The interactions are subject to the type of healthcare and professionals’ practices. The findings show that at least two contexts or settings could be identified for intervention: specialist care or chronic illness care. These pathways are worth further exploring to form engagement strategies that have the potential to improve health outcomes and healthcare quality. Secondly, this network approach is a method that policy makers can use to assess the extent of influence between patient engagement measures or to understand potential conflicts between organizational priorities and engagement activities. Competing priorities can be found in organizations or systems[12] and we think the understanding of the interdependencies or connectedness between variables through network models would be helpful to assess some of the concerns. With enough resources, the identified interactions between engagement measures can be verified with randomized trials or other studies.

For policy makers, the implementation strategies will rely on the policy objectives and resources. For example, providing clear instructions is directly linked to patient engagement in both chronic illness and mental health care. If the policy aims to involve patients as much as possible in chronic illness care, the formation of treatment plans may be the target of intervention. This is to focus on the immediate factors, if causation confirmed. In contrast, the opposite approach is to focus on the factors that are linked to many potential outcomes through several intermediate variables.[40] These non-immediate or distant factors may possess more extensive influence over patient engagement through multiple channels. In the same example, a policy focusing on how health professionals give patients instruction has the potential to influence engagement in chronic care and mental health.

#### Future work

There are also opportunities for future research. First, we take advantage of the random samples from the 2014 IHPS and do not adopt the complex survey design in the 2014 IHPS for the lack of the adequate statistical tool[41]. The adjustment of complex survey design to report nationally representative statistics will be an important opportunity to advance our understanding in patient engagement. Second, we are unaware of which mechanisms, whether knowledge enhancement, skill development, patient confidence, or behavioural change[37], the associations between variables are built upon. We suggest adopting more theory-based variables for future surveys in order to test these possible theories or mechanisms.

### Strengths and limitations

The strengths of this study include the use of comparable interview methods across countries, a large sample size, and models adequate for network analysis. The inclusion of patient engagement measures enables us to understand the potential role of engagement in health improvement. However, there are limitations to this study. This is a secondary data analysis using observational and cross-sectional data. The directions between nodes or variables cannot be seen as causal[42]. Second, the data cleaning policies are consistent with a previous publication[14] and require assumptions. For example, the “don’t know” category is recoded as missing. The importance of these assumptions needs to be tested in the future. Third, the 2014 IHPS was the first one to have patient engagement variables.[14] It is not possible to understand the trends in patient engagement in the included countries. Fourth, there are other patient engagement theories or research approaches that have been developed and reviewed recently.[4, 6, 7] The theoretic framework of the 2014 IHPS may not be satisfying when this study is published. Fifth, there are very limited numbers of health outcomes for study in the 2014 IHPS, including mental problems and self-rated health status. Lastly, there are other dimensions of patient engagement or outcomes that are not surveyed in the 2014 IHPS, such as health literacy, collaboration between patients and physicians, and self-management skills.[37, 43] We are not certain about the role of these unmeasured dimensions.

## Conclusion

We apply Bayesian network modeling to show the interactions between contextual factors and variables of patient engagement based on the information from an international survey which is being referred to as 2014 IHPS. Network modeling is useful to integrate expert knowledge and observational data. We show that patient engagement is conditionally dependent on types of care and that there are variables associated with both health status and patient engagement. Our analysis shows that there may be at least two clinical settings to engage patients in their care, chronic illness or specialist care. The individual characteristics and types of care are not necessarily linked to patient engagement variables. Our empirical findings can help in refining the policy objectives and in searching priorities for systemic interventions. We suggest engaging patients in their healthcare by providing written plans and actively communicating with patients for chronic illnesses, as well as encouraging specialists to provide treatment options.

## Supporting information

S1 Appendix The characteristics of the variables in the 2014 Commonwealth Fund International Health Policy Survey. (XLSX)

S2 Appendix The networks of the variables in the 2014 Commonwealth Fund International Health Policy Survey, description of variables labeled. (PDF)

S3 Appendix The networks of the variables in the 2014 Commonwealth Fund International Health Policy Survey, variable names labeled. (PDF)

S4 Appendix The networks of the variables in the 2014 Commonwealth Fund International Health Policy Survey, short names labeled. (PDF)

S5 Appendix The probability distribution of the measures of patient engagement or support in chronic illness care. (DOCX)

S6 Appendix The probability distributions of patient engagement or support in specialist care. (DOCX)
