# Supplementary material for: A network perspective of engaging patients in specialist and chronic illness care: The 2014 International Health Policy Survey
Source: PLoS One. 2018 Aug 13;13(8):e0201355. doi: 10.1371/journal.pone.0201355 (PMC6089423; doi:10.1371/journal.pone.0201355)
Supplement: S2 Appendix — (PDF) [file pone.0201355.s002.pdf]

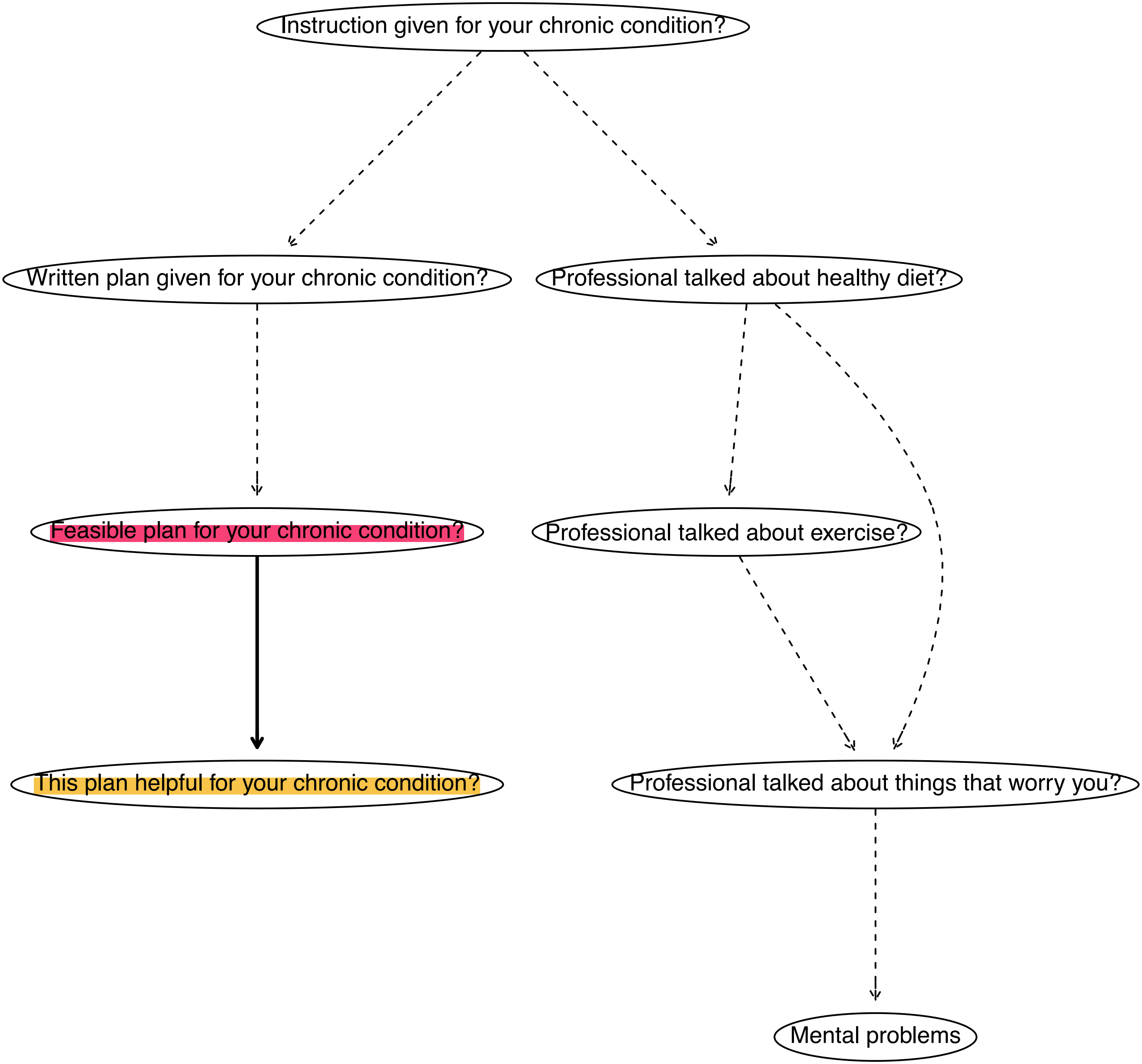
Instruction given for your chronic condition?
Written plan given for your chronic condition?
Professional talked about healthy diet?
Feasible plan for your chronic condition?
Professional talked about exercise?
This plan helpful for your chronic condition?
Professional talked about things that worry you?
Mental problems

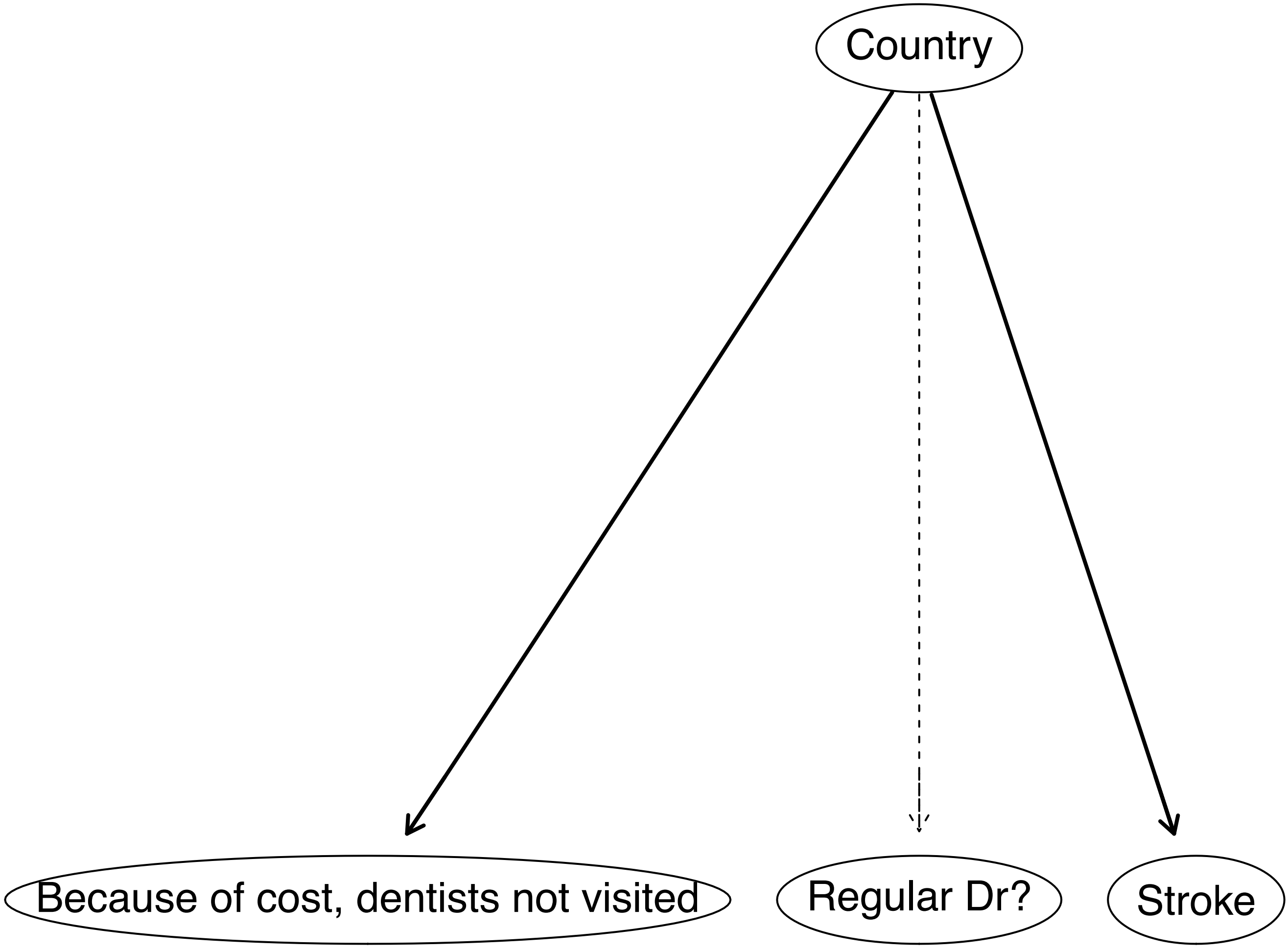
Country
Because of cost, dentists not visited
Regular Dr?
Stroke

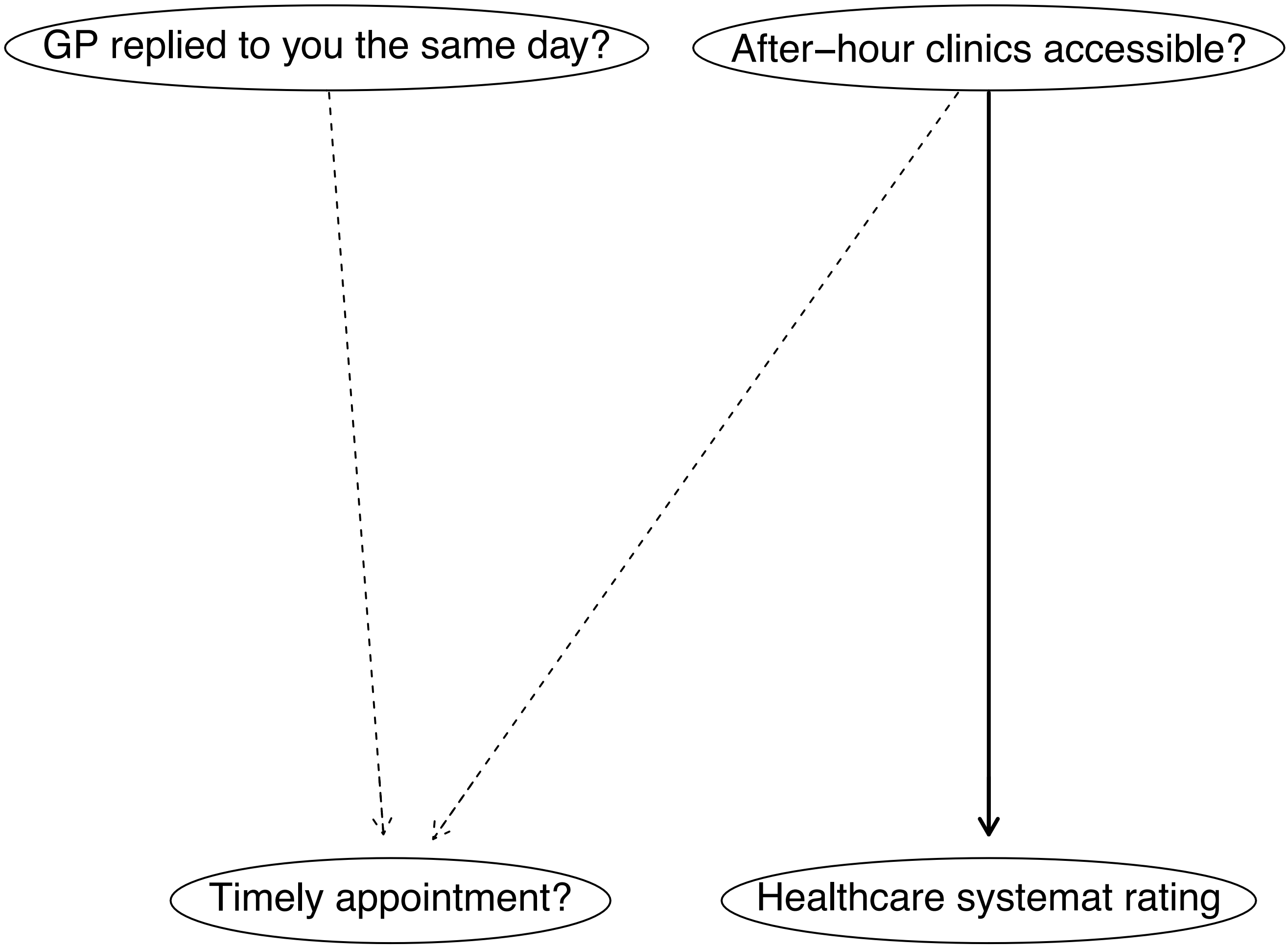
GP replied to you the same day?
After−hour clinics accessible?
Timely appointment?
Healthcare systemat rating

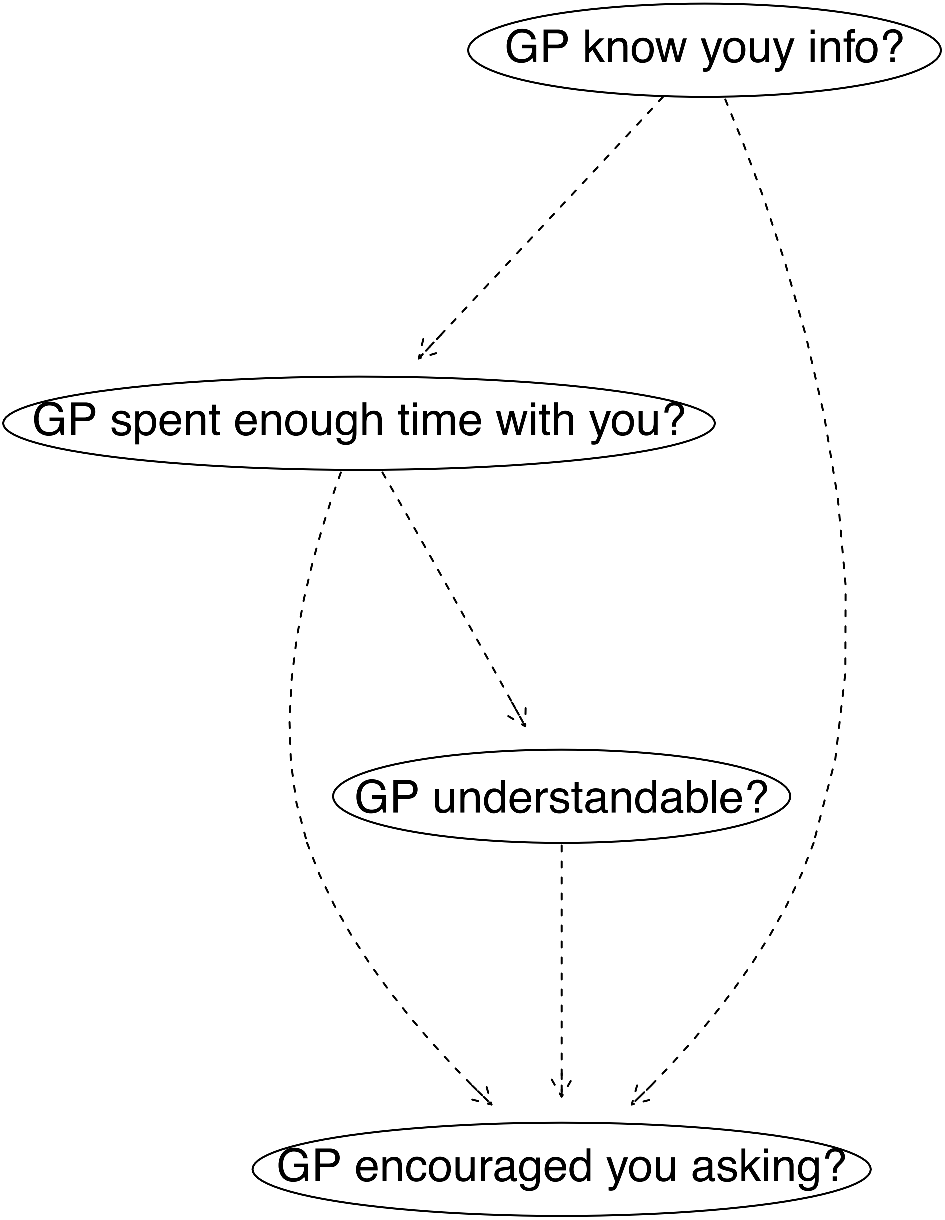
GP know youy info?
GP spent enough time with you?
GP understandable?
GP encouraged you asking?

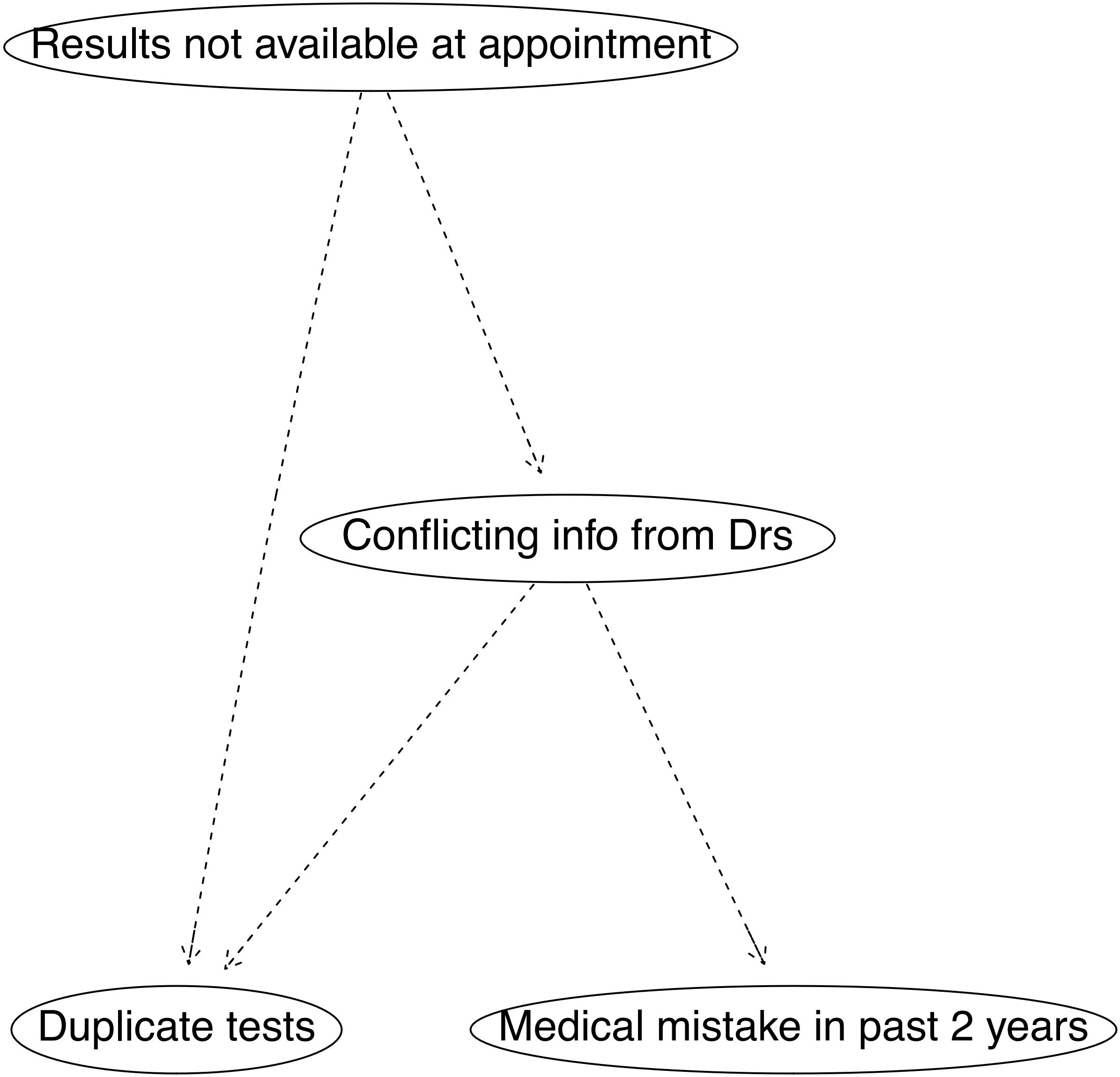
Results not available at appointment
Conflicting info from Drs
Duplicate tests
Medical mistake in past 2 years

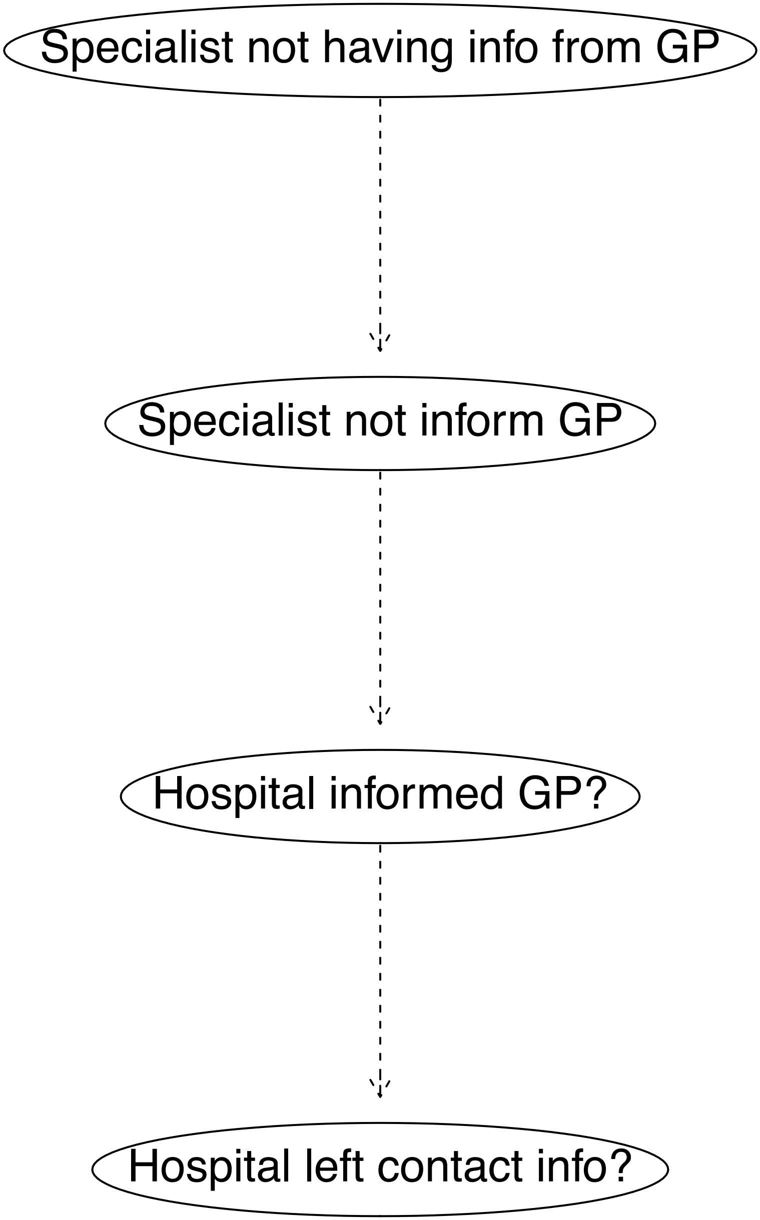
Specialist not having info from GP
Specialist not inform GP
Hospital informed GP?
Hospital left contact info?

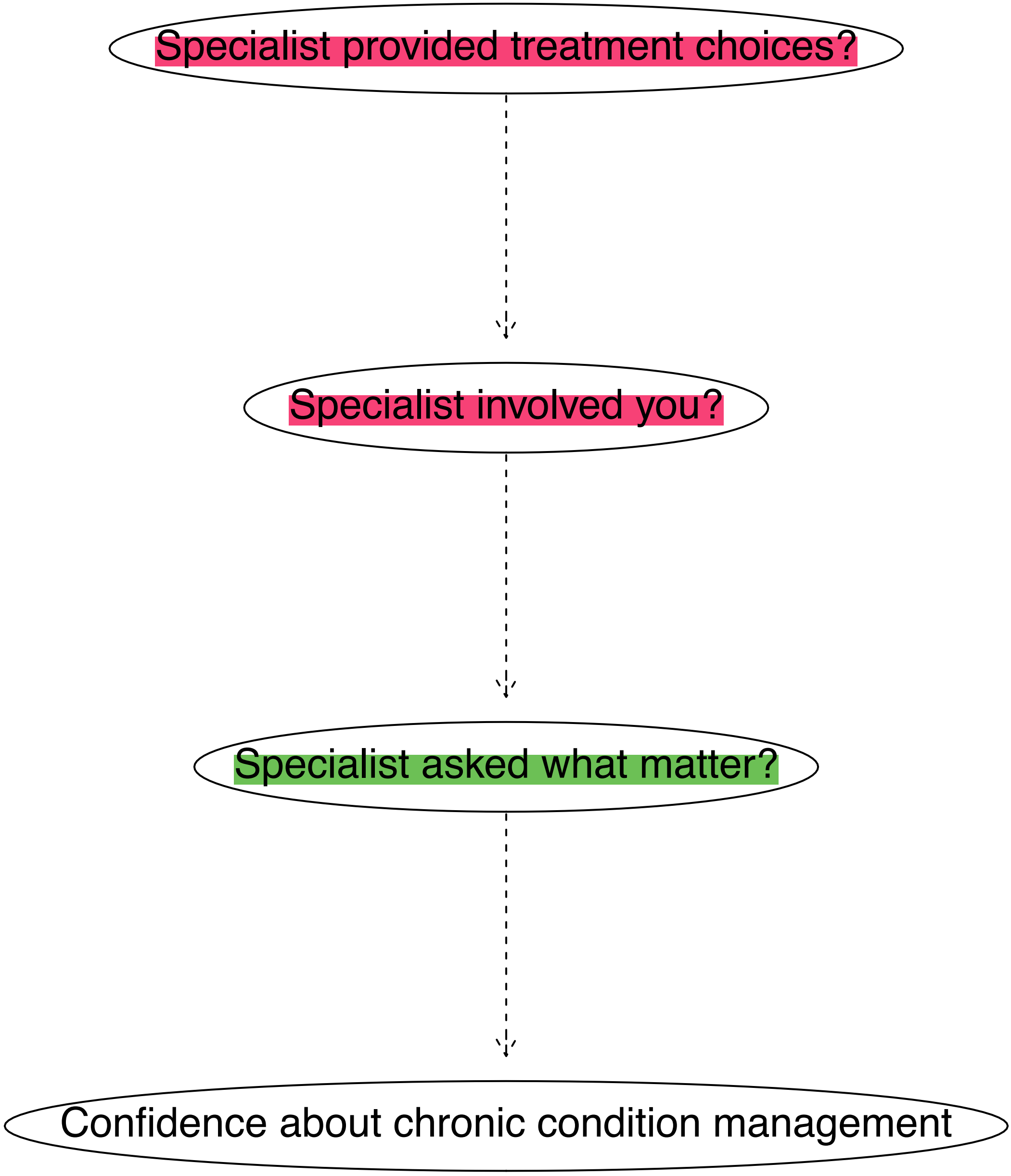
Specialist provided treatment choices?
Specialist involved you?
Specialist asked what matter?
Confidence about chronic condition management

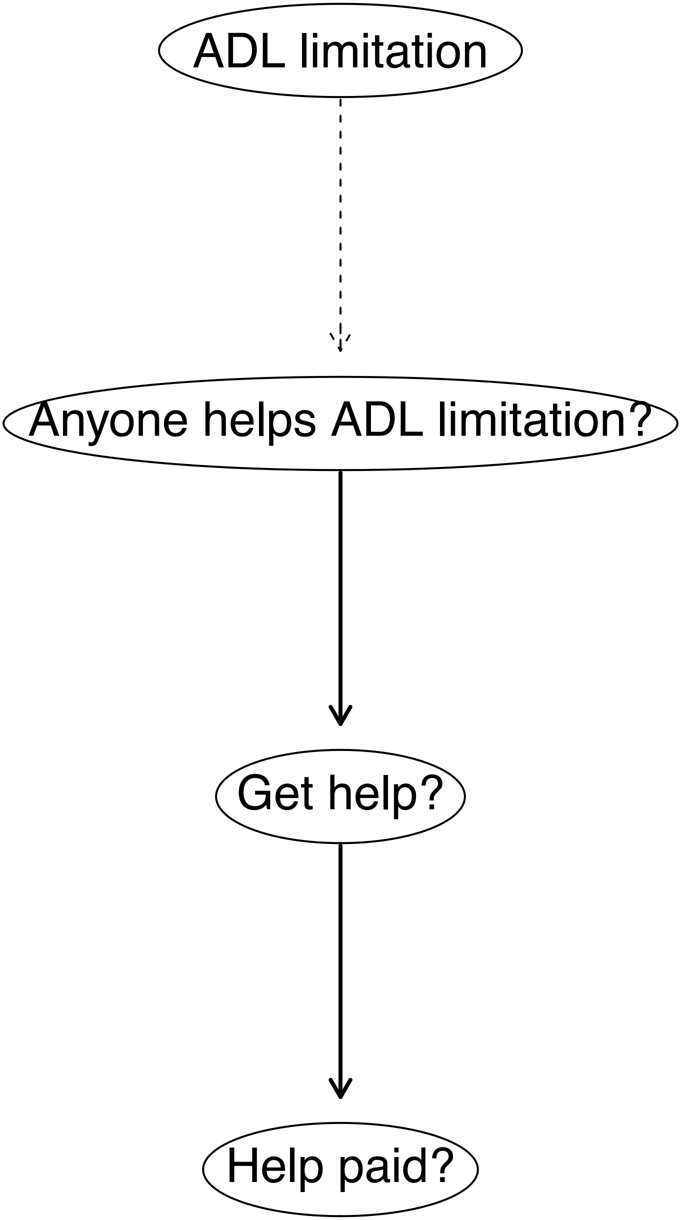
ADL limitation
Anyone helps ADL limitation?
Get help?
Help paid?

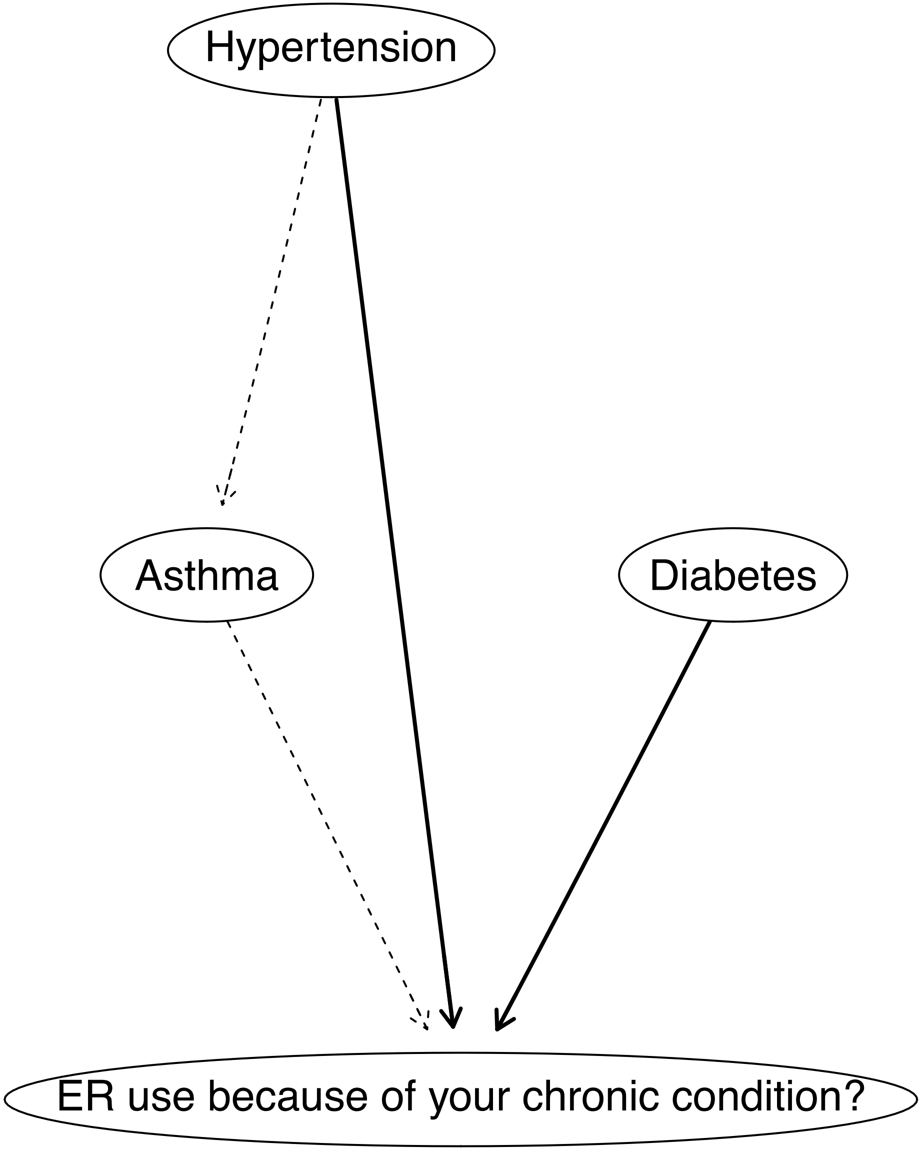
Hypertension
Asthma
Diabetes
ER use because of your chronic condition?

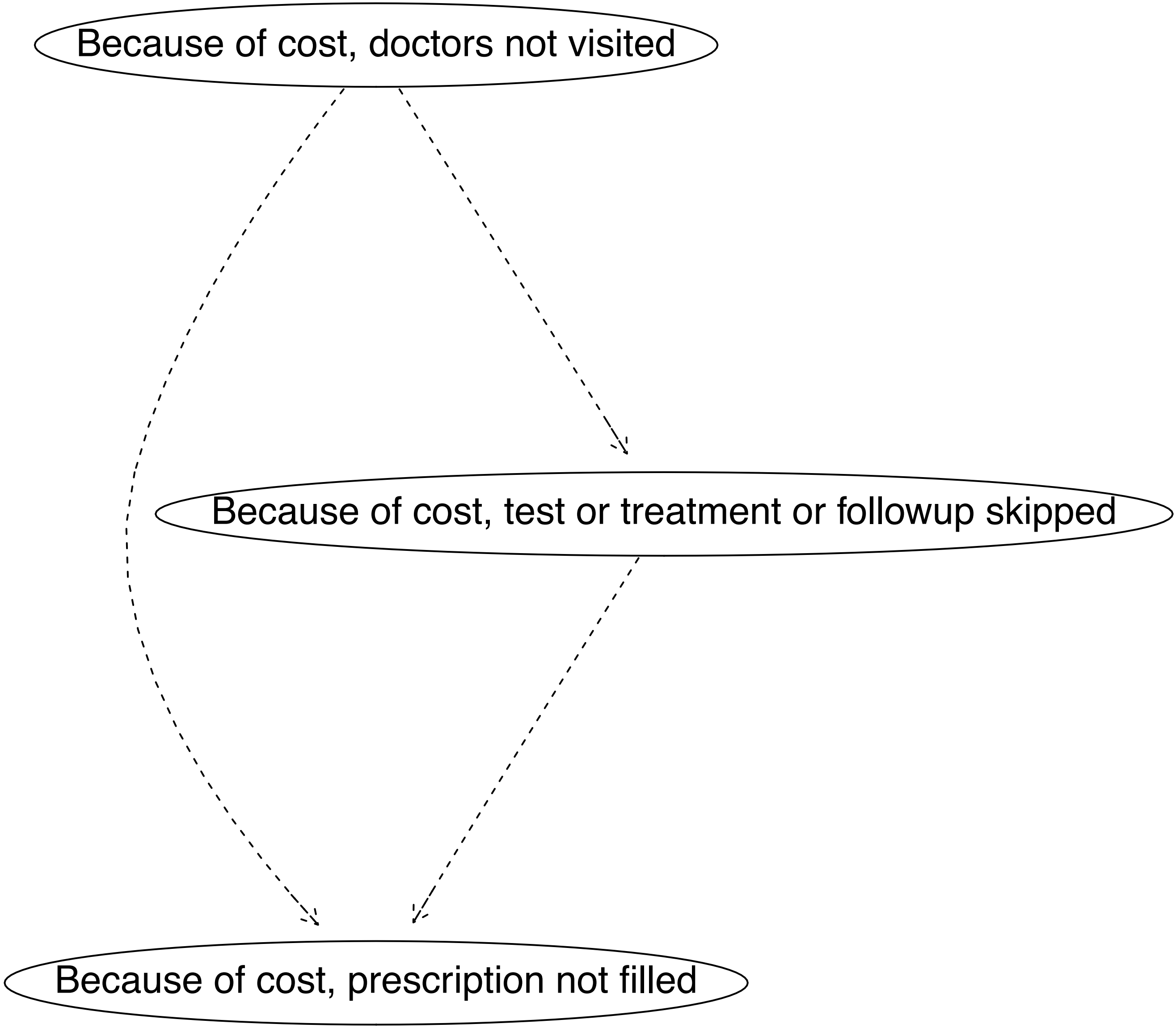
Because of cost, doctors not visited
Because of cost, test or treatment or followup skipped
Because of cost, prescription not filled

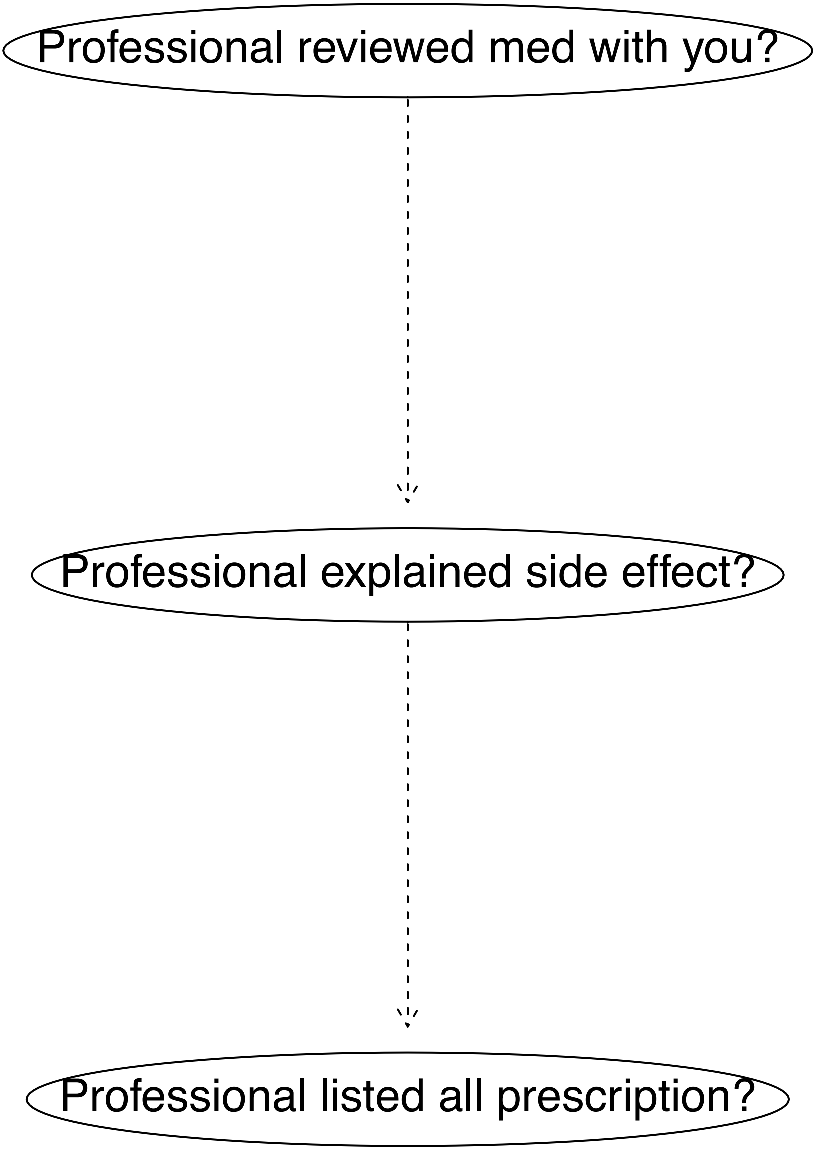
Professional reviewed med with you?
Professional explained side effect?
Professional listed all prescription?

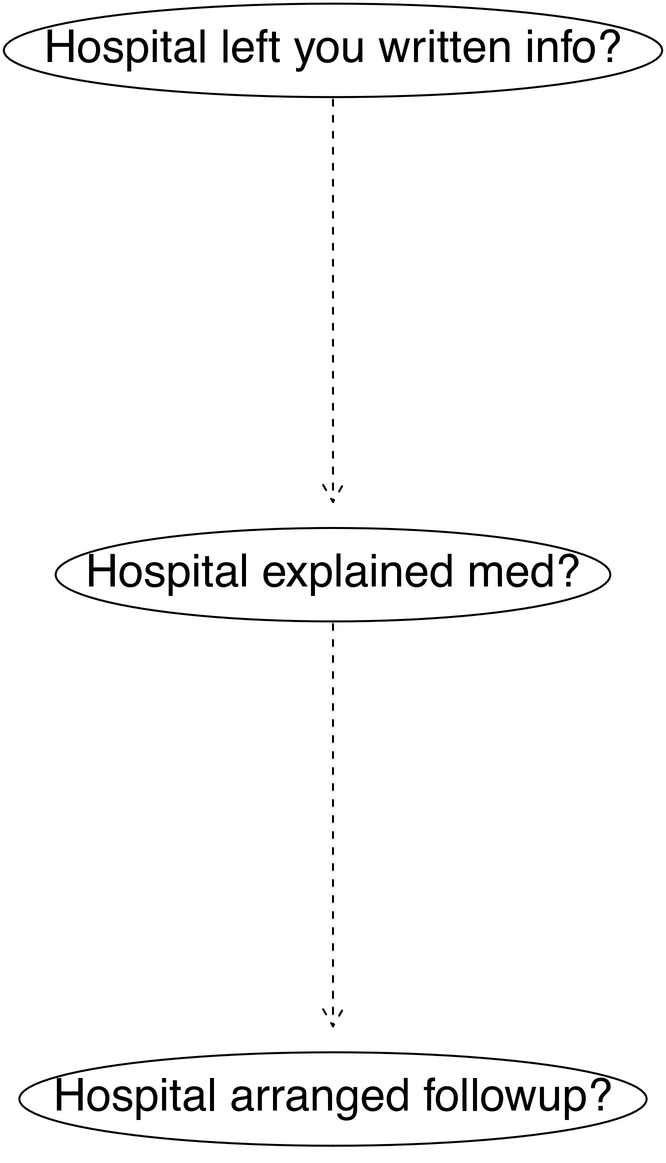
Hospital left you written info?
Hospital explained med?
Hospital arranged followup?

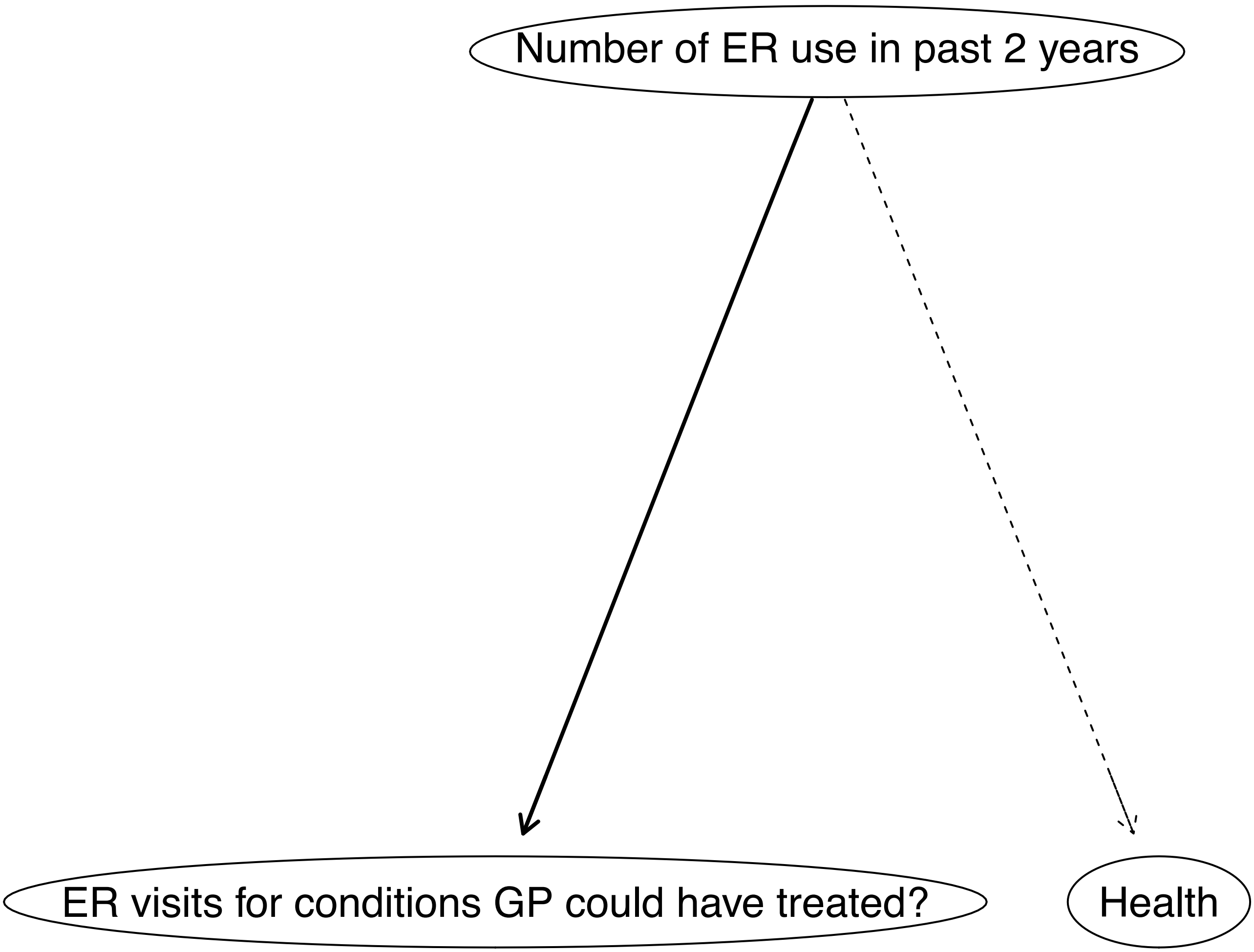
Number of ER use in past 2 years
ER visits for conditions GP could have treated?
Health

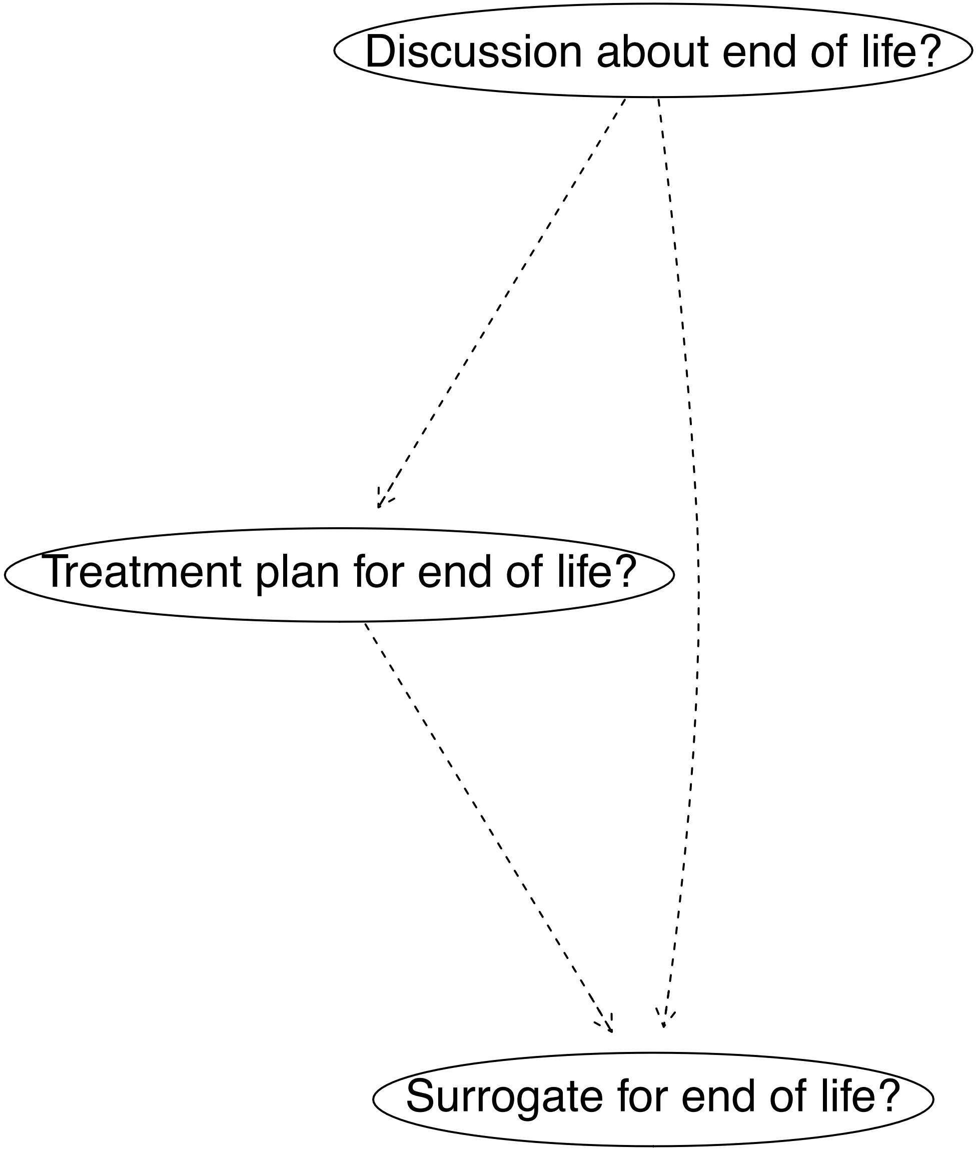
Discussion about end of life?
Treatment plan for end of life?
Surrogate for end of life?

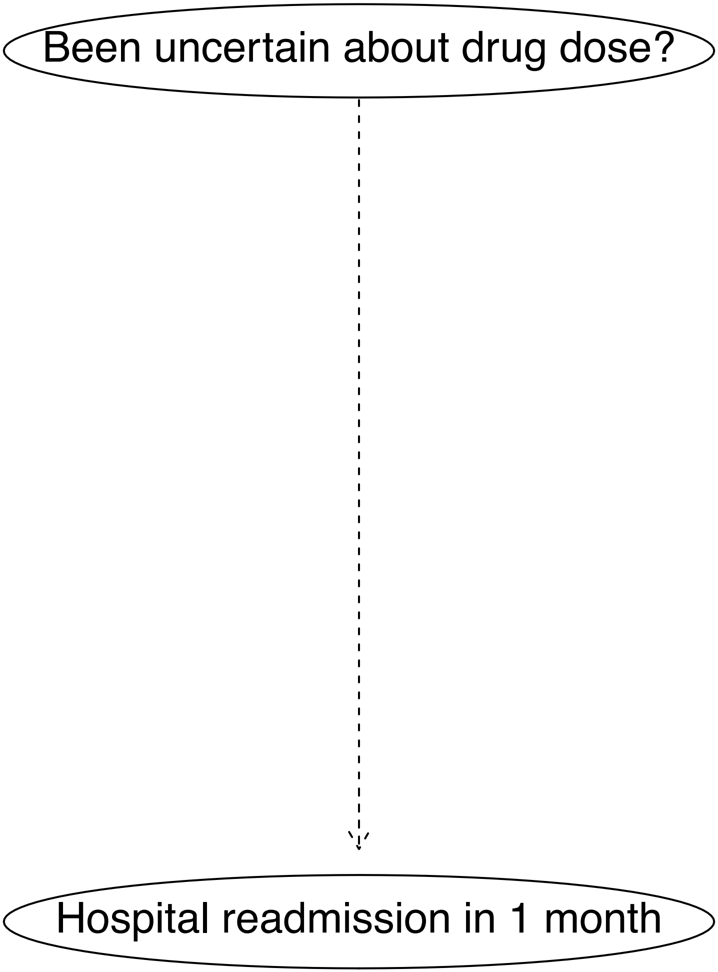
Been uncertain about drug dose?
Hospital readmission in 1 month

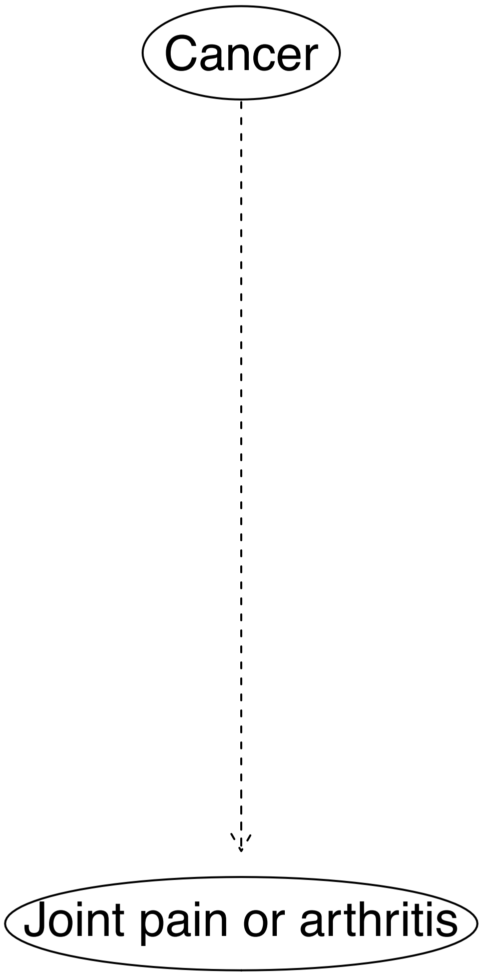
Cancer
Joint pain or arthritis

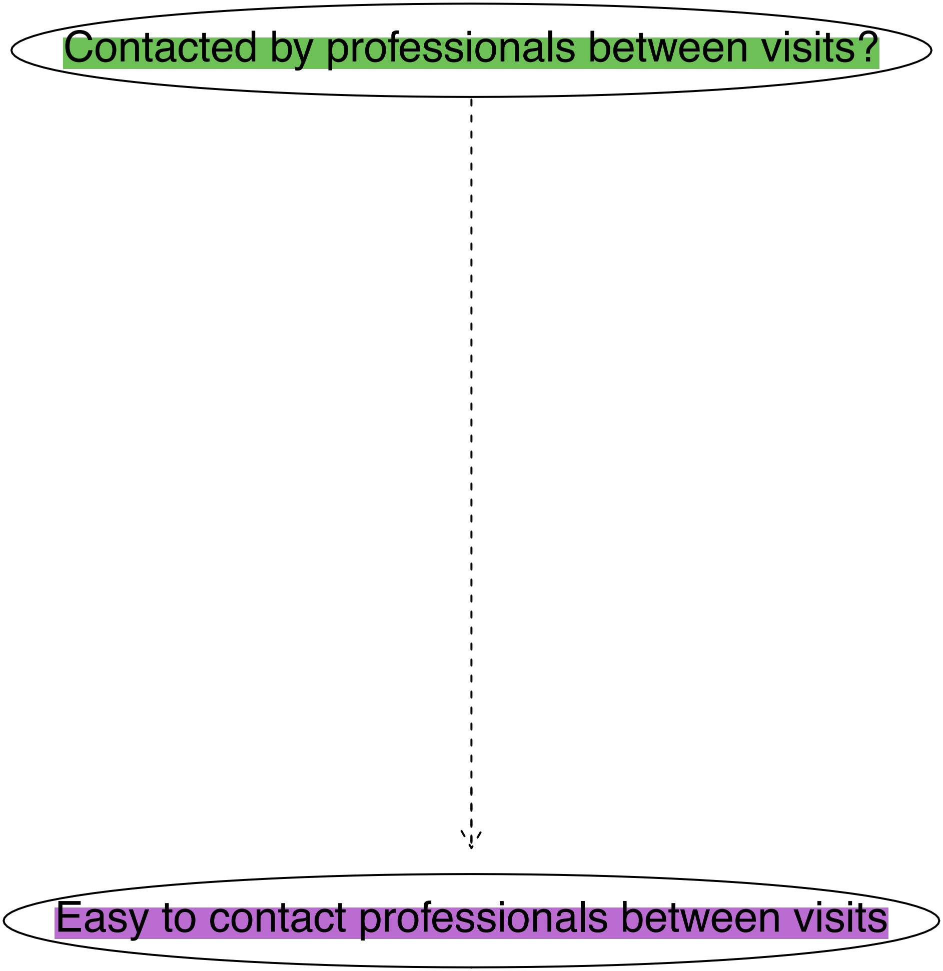

Contacted by professionals between visits?
Easy to contact professionals between visits

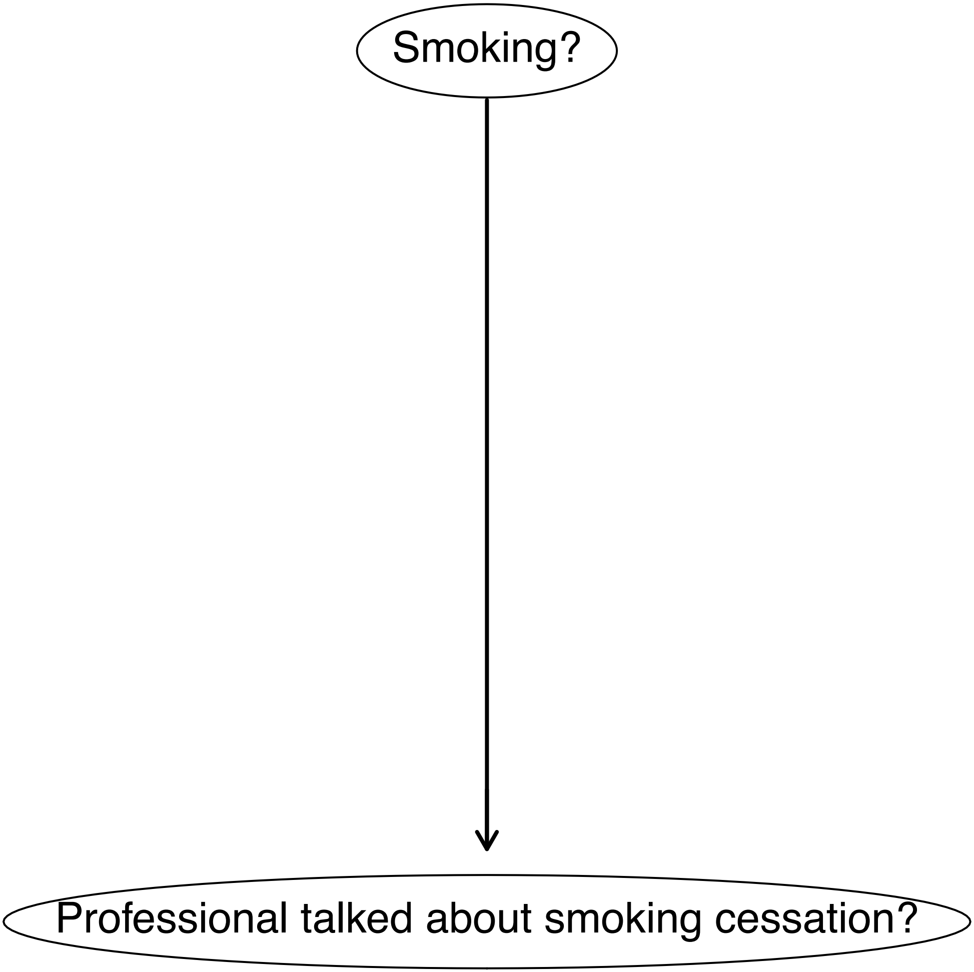

Smoking?
Professional talked about smoking cessation?

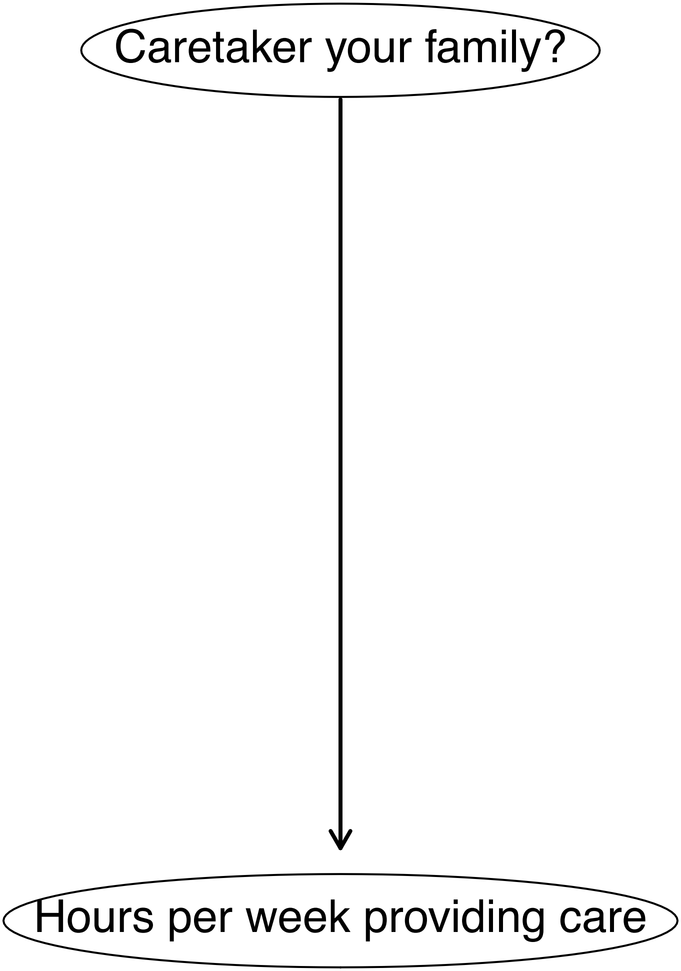

Caretaker your family?
Hours per week providing care
