# Supplementary figures and images for: A network perspective of engaging patients in specialist and chronic illness care: The 2014 International Health Policy Survey
Source: PLoS One. 2018 Aug 13;13(8):e0201355. doi: 10.1371/journal.pone.0201355 (PMC6089423; doi:10.1371/journal.pone.0201355)

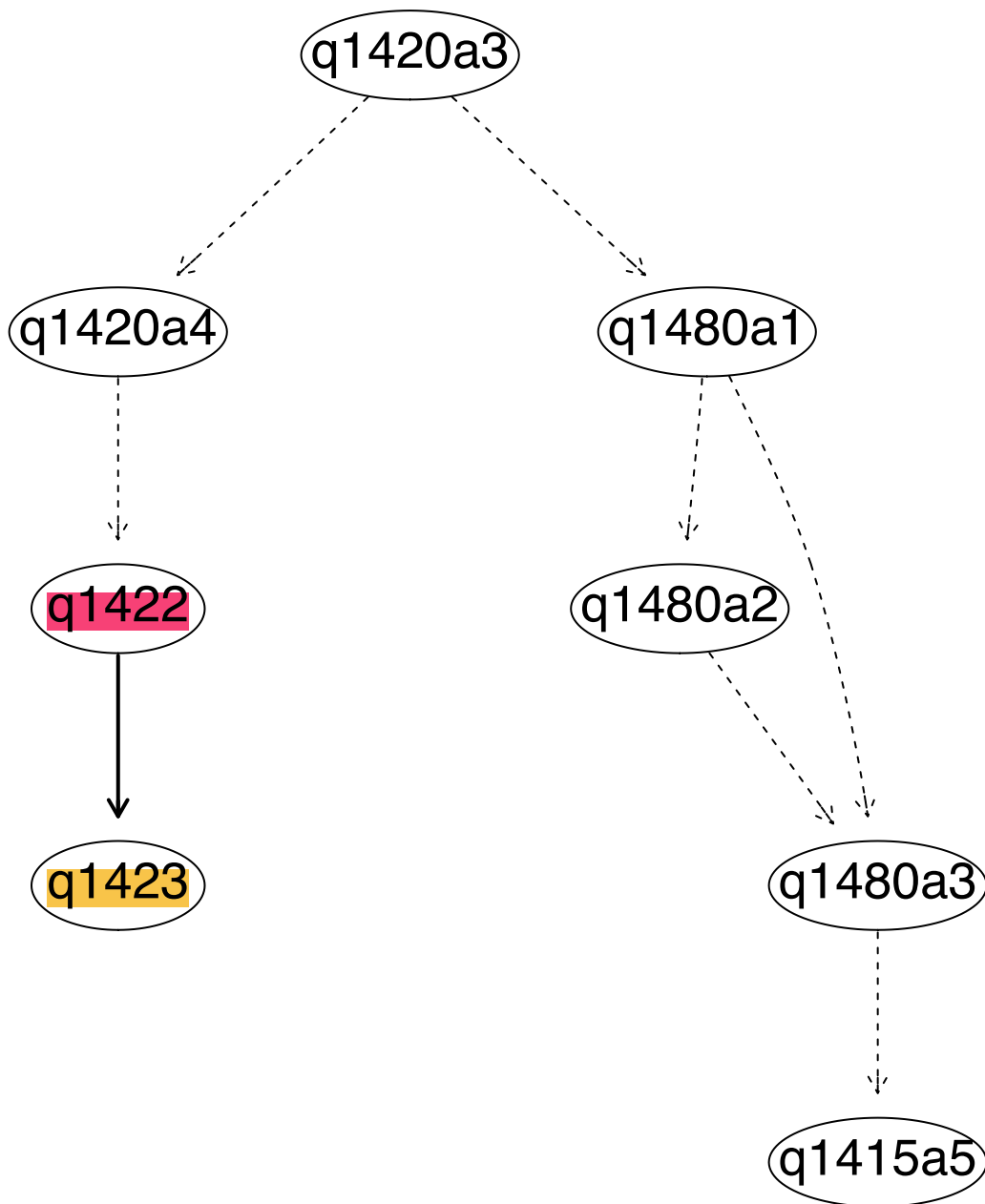

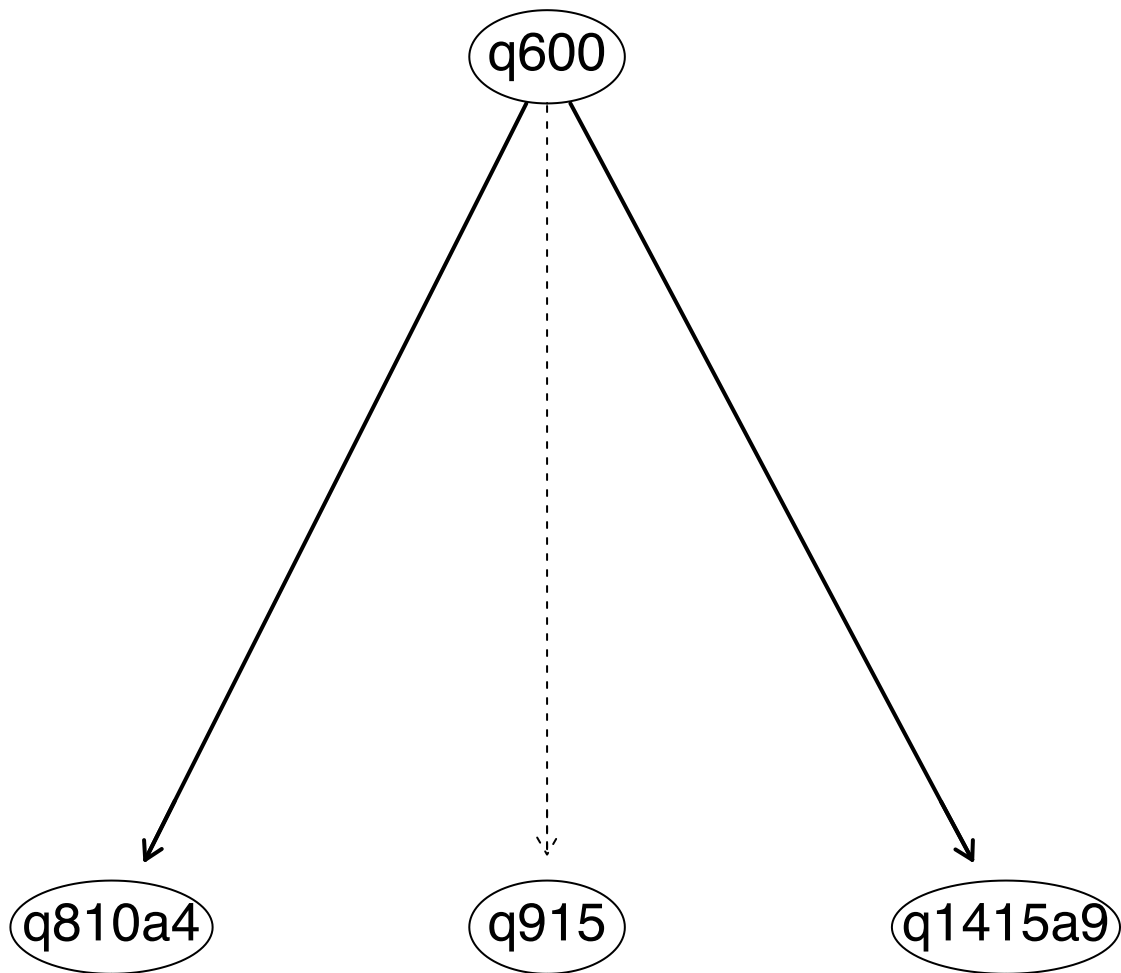

q935

q815

q820

q1621

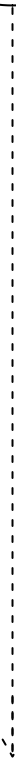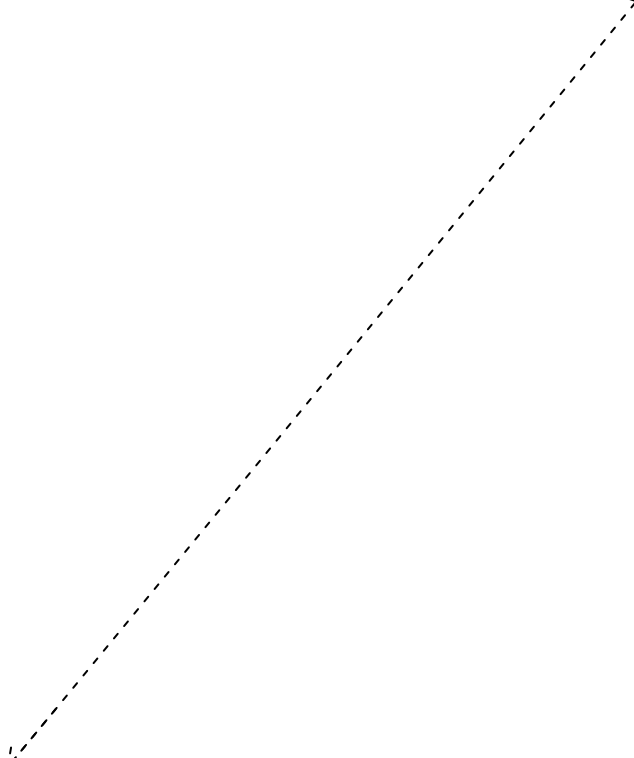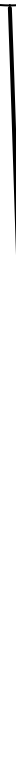

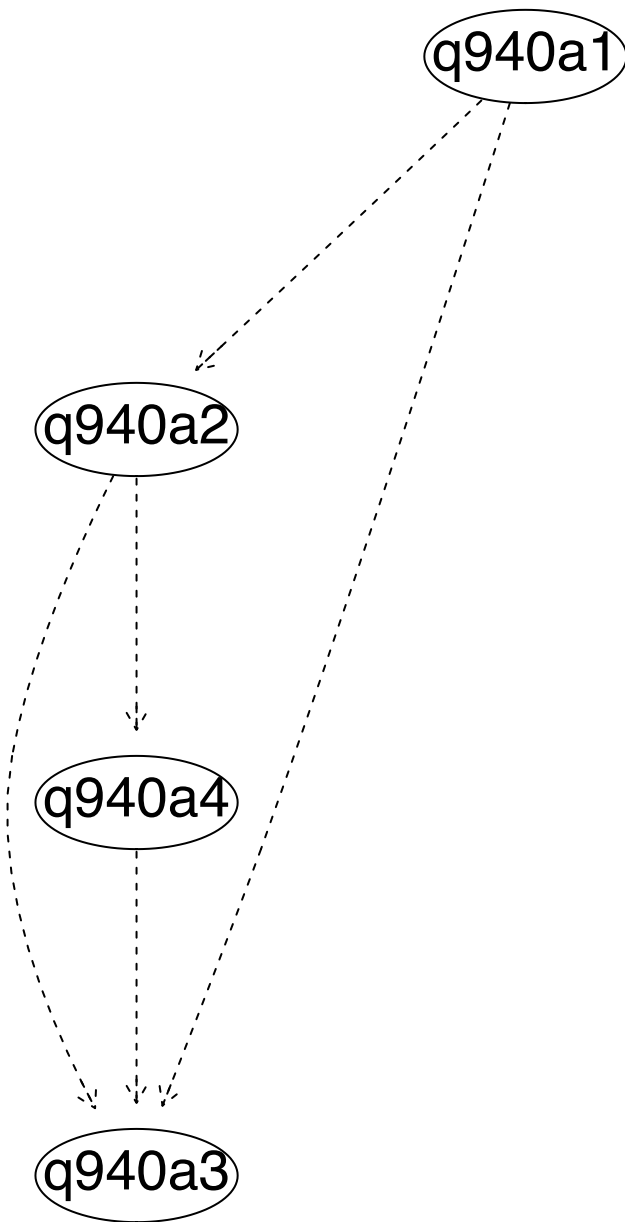

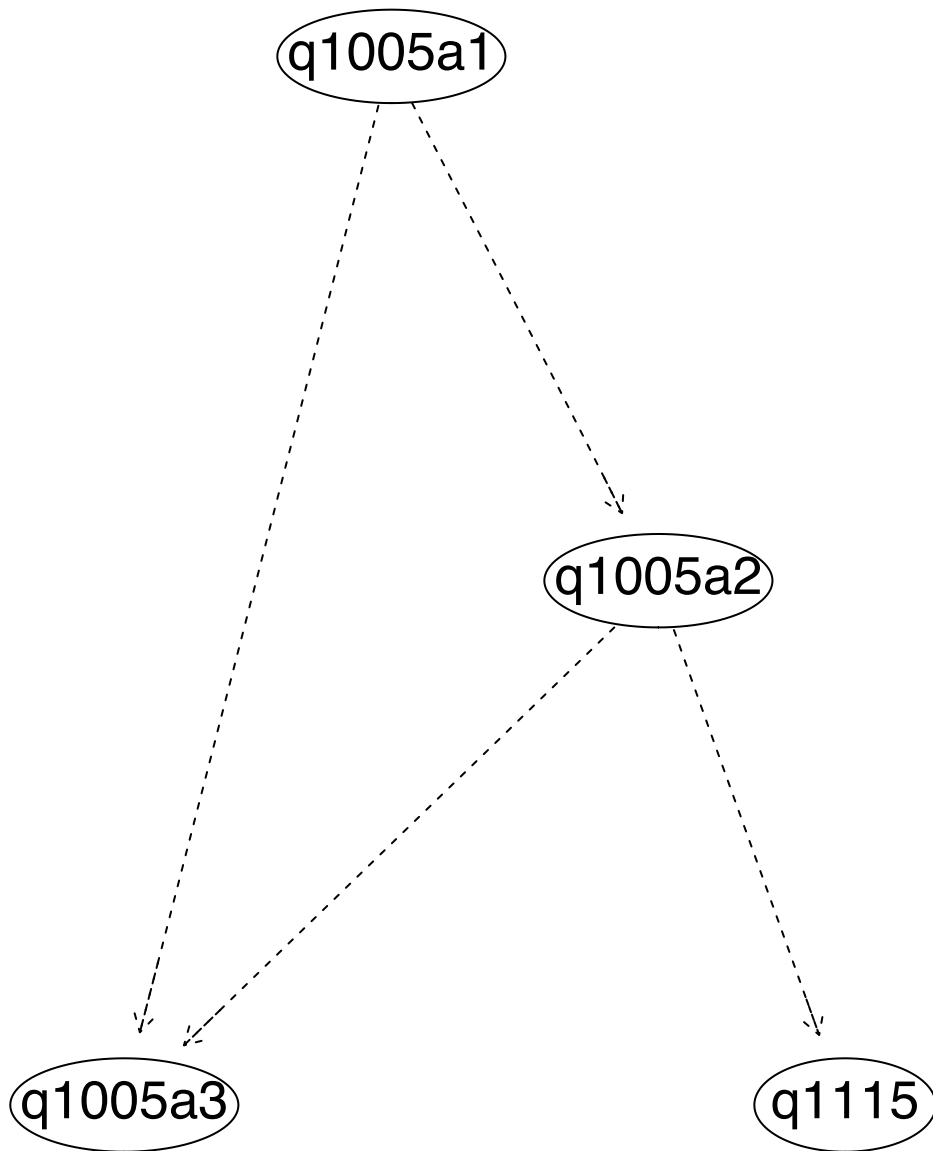

q1050a1

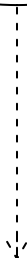

q1050a2

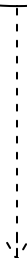

q1325

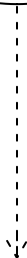

q1310a4

q1065a1

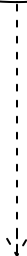

q1065a2

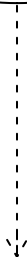

q1065a3

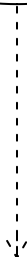

q1424

q1402

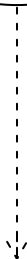

q1405

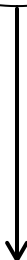

q1407

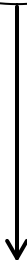

q1408

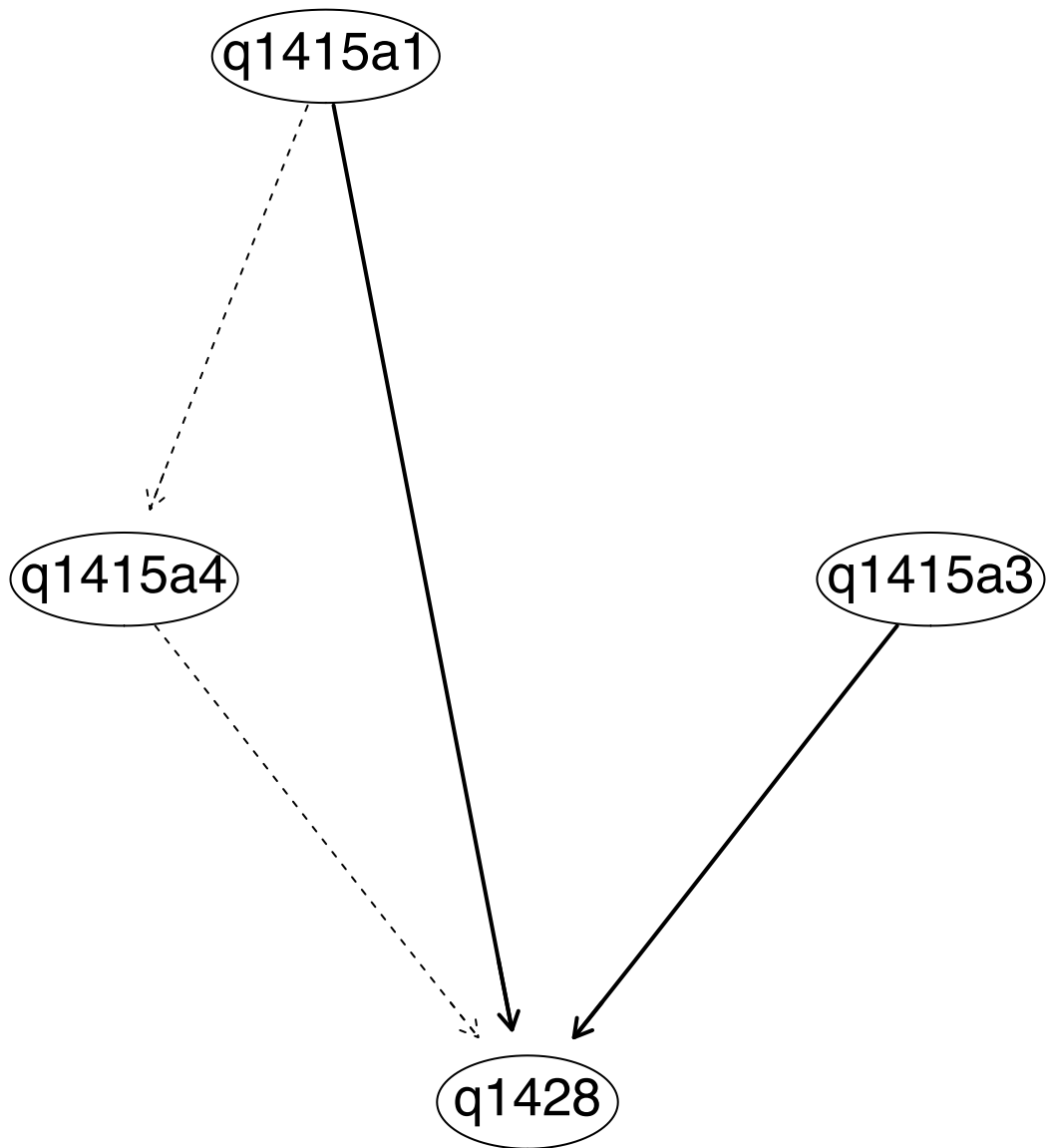

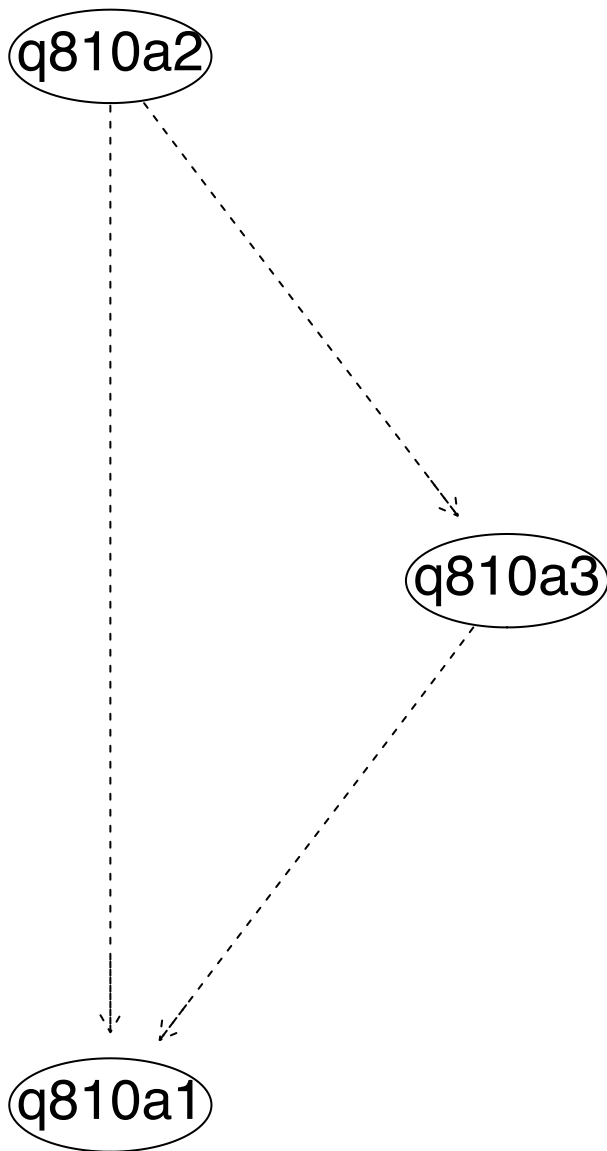

q1110a1

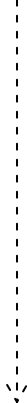

q1110a2

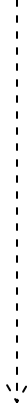

q1110a3

q1310a1

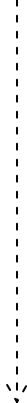

q1310a3

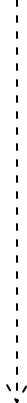

q1310a2

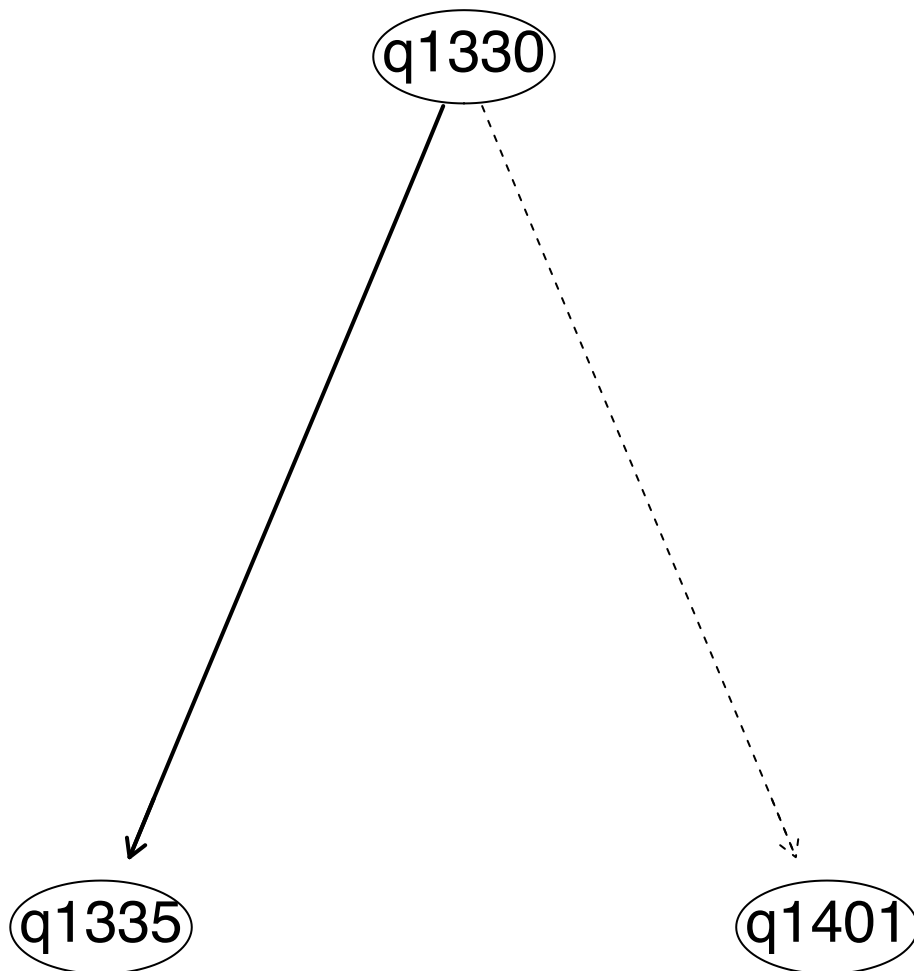

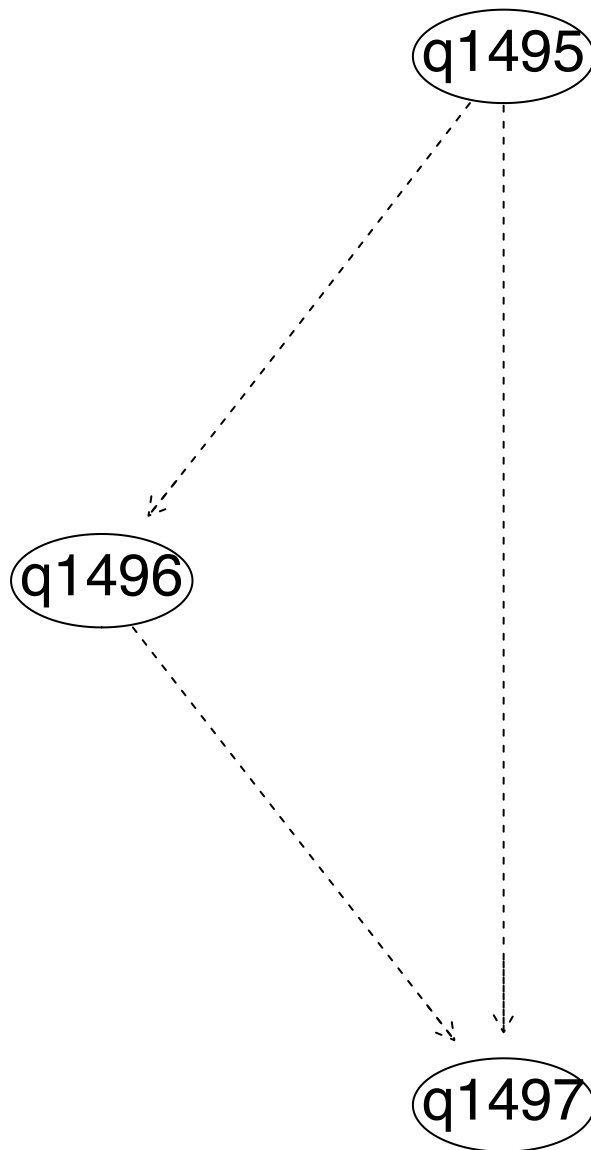

q1135

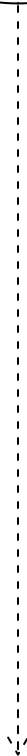

q1315

q1415a6

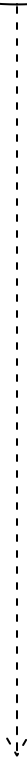

q1415a8

q1425a1

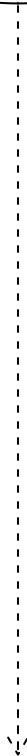

q1425a2

q1483

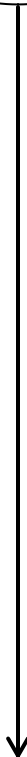

q1485

q1492

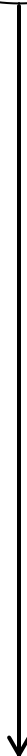

q1494

Supplement: S3 Appendix — (PDF) [file pone.0201355.s003.pdf]
