# Supplementary material for: A network perspective of engaging patients in specialist and chronic illness care: The 2014 International Health Policy Survey
Source: PLoS One. 2018 Aug 13;13(8):e0201355. doi: 10.1371/journal.pone.0201355 (PMC6089423; doi:10.1371/journal.pone.0201355)
Supplement: S4 Appendix — (PDF) [file pone.0201355.s004.pdf]

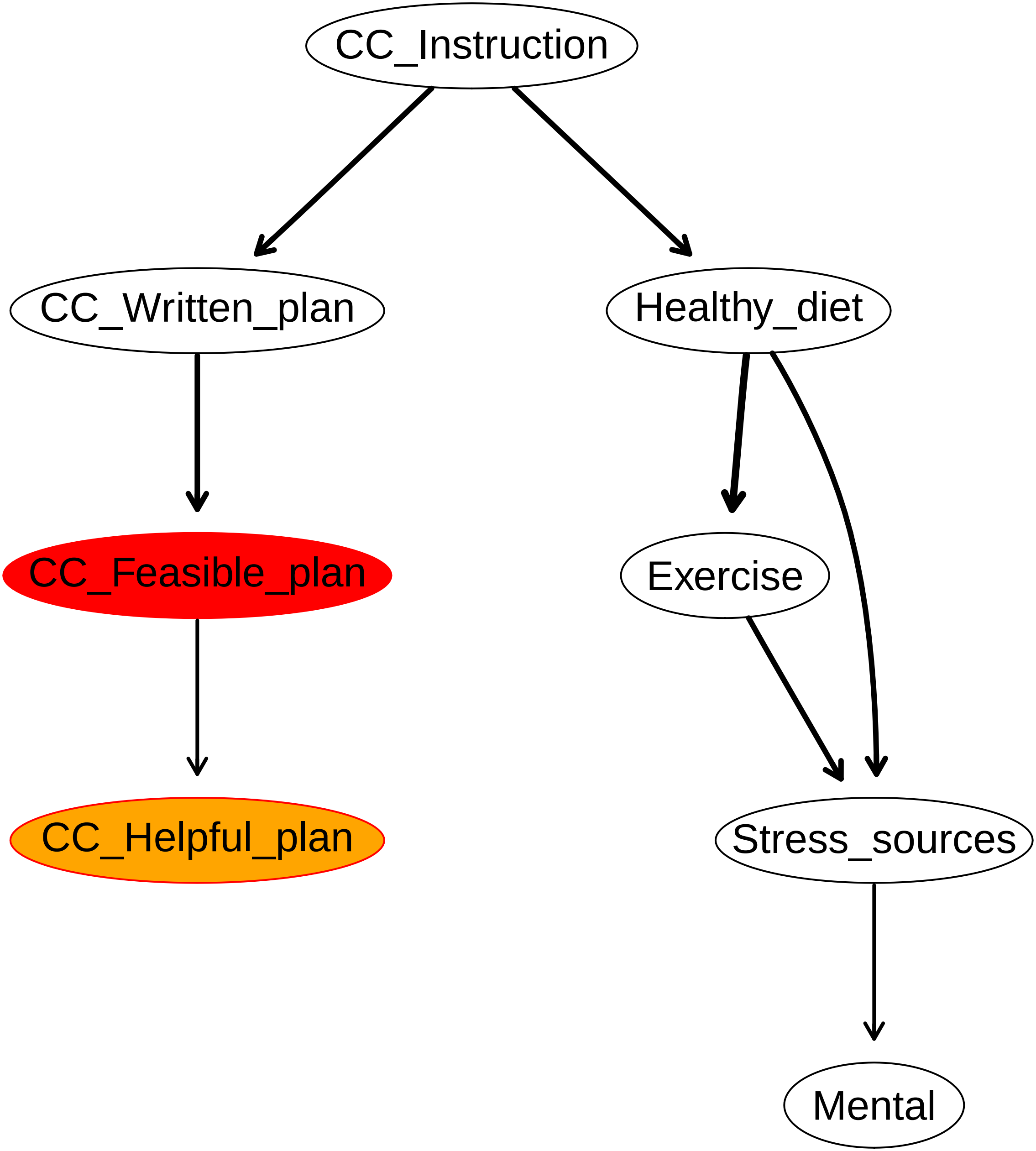
CC_Instruction
CC_Written_plan
Healthy_diet
CC_Feasible_plan
Exercise
CC_Helpful_plan
Stress_sources
Mental

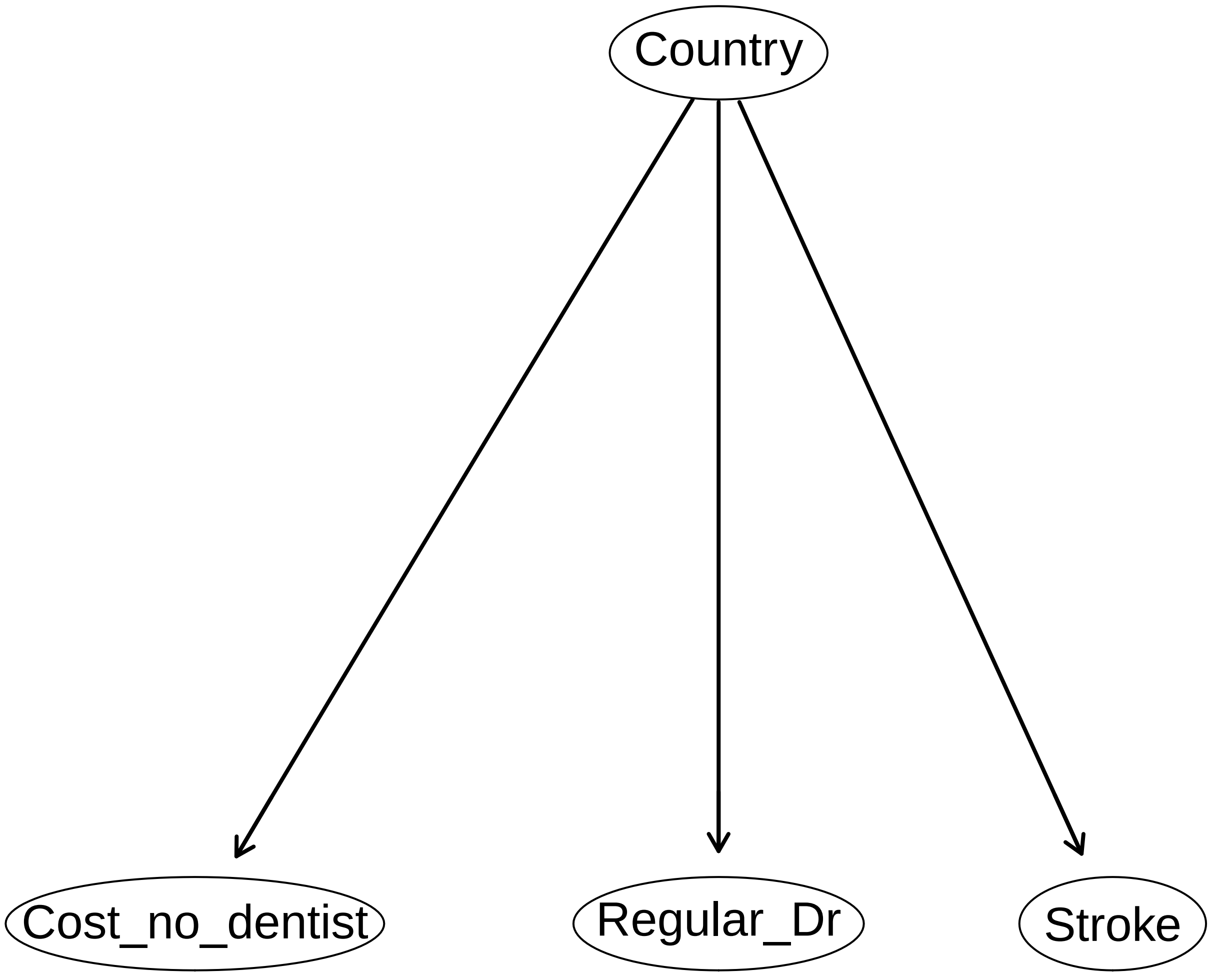
Country
Cost_no_dentist
Regular_Dr
Stroke

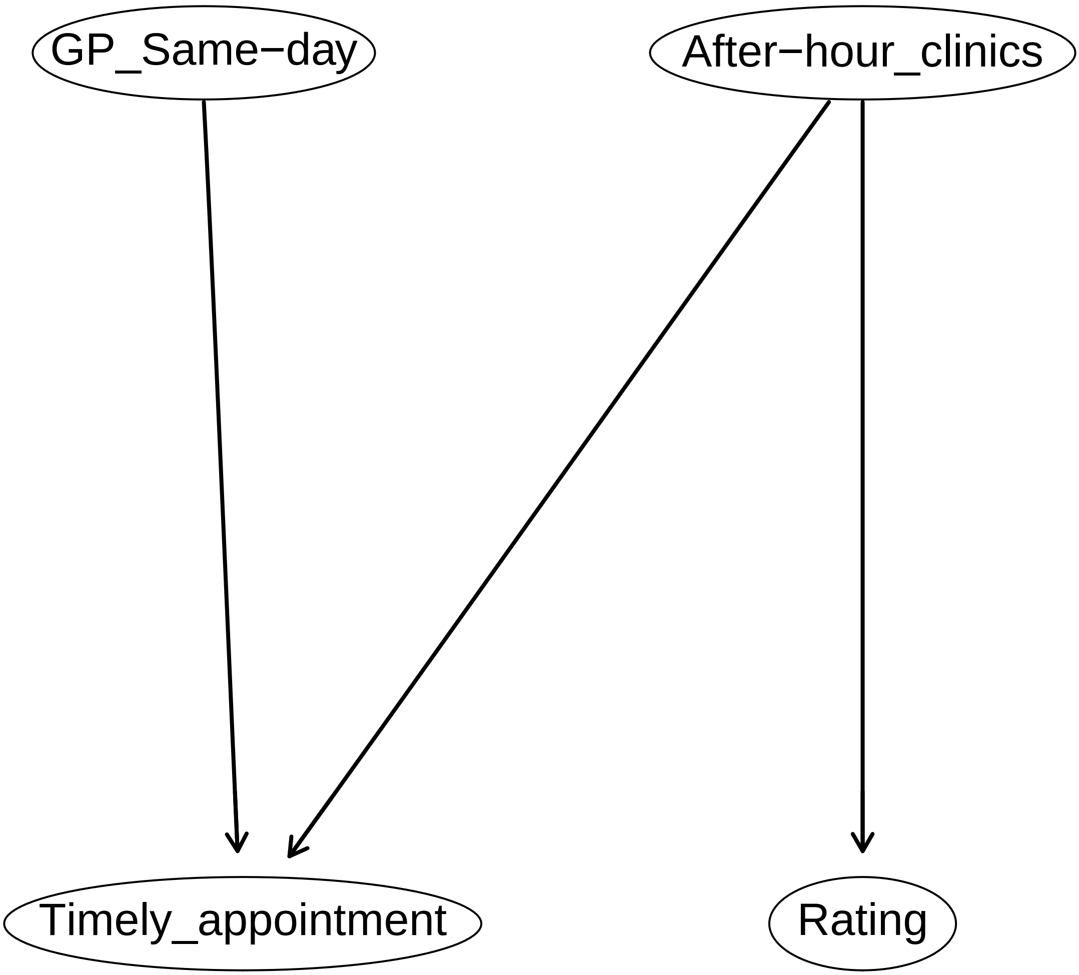
GP_Same−day
After−hour_clinics
Timely_appointment
Rating

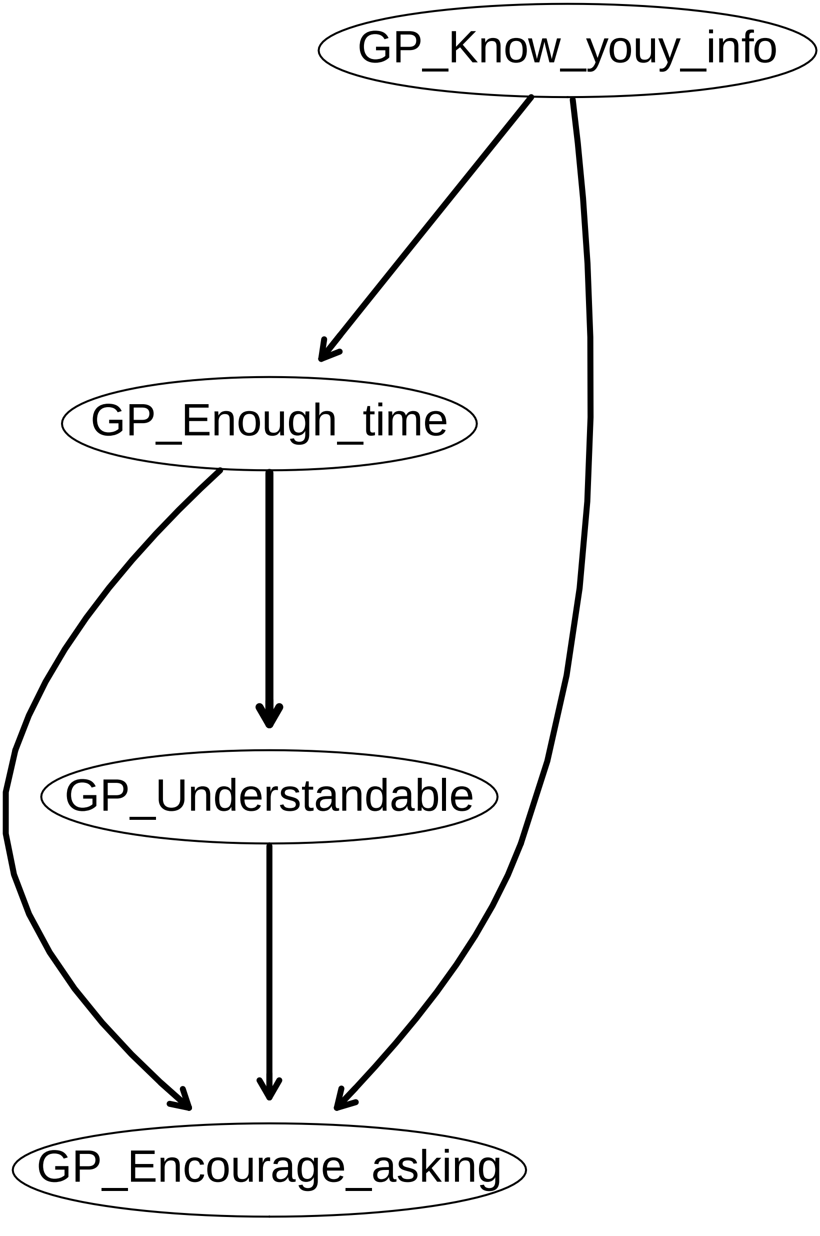
GP_Know_youy_info
GP_Enough_time
GP_Understandable
GP_Encourage_asking

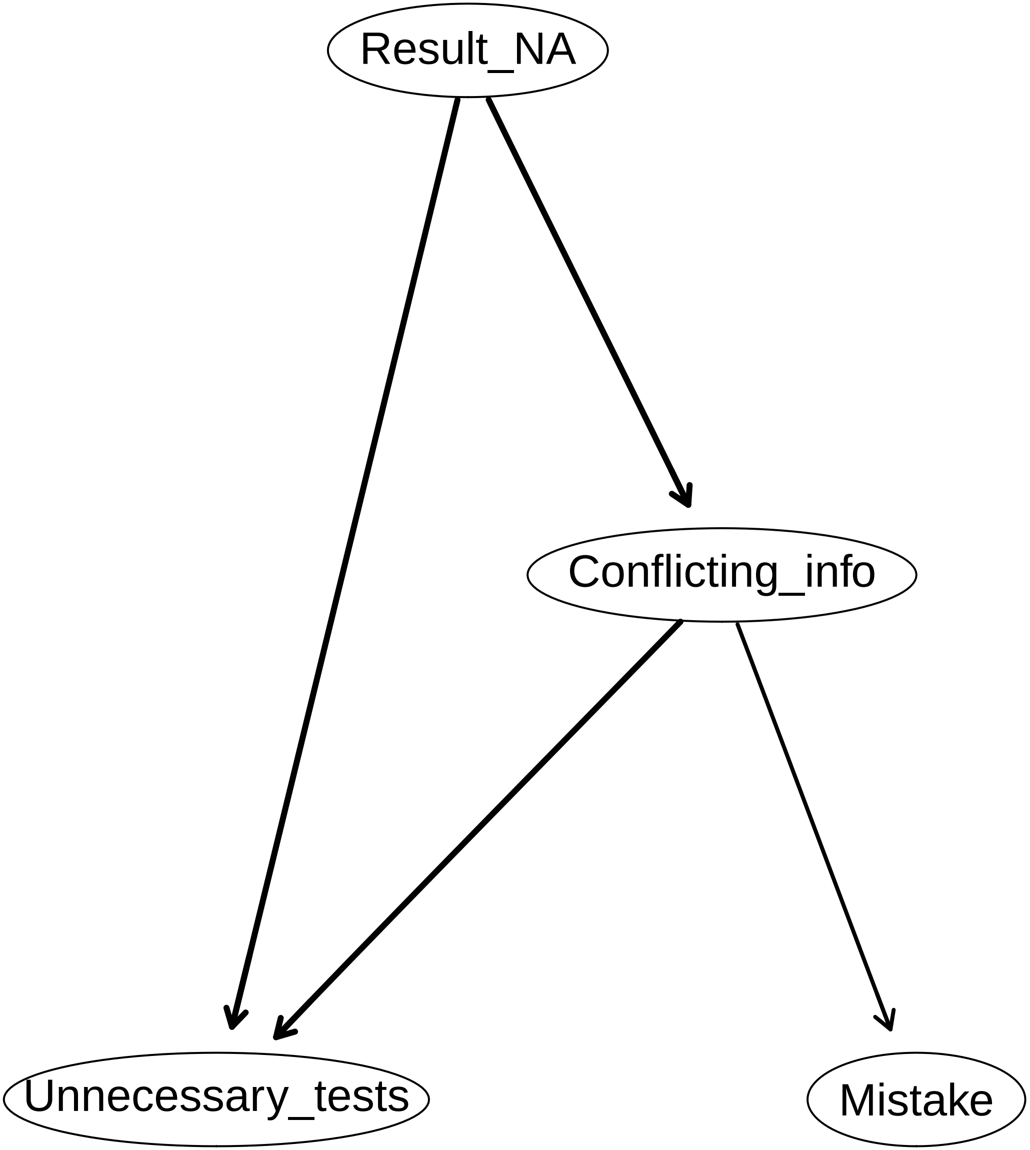
Result_NA
Conflicting_info
Unnecessary_tests
Mistake

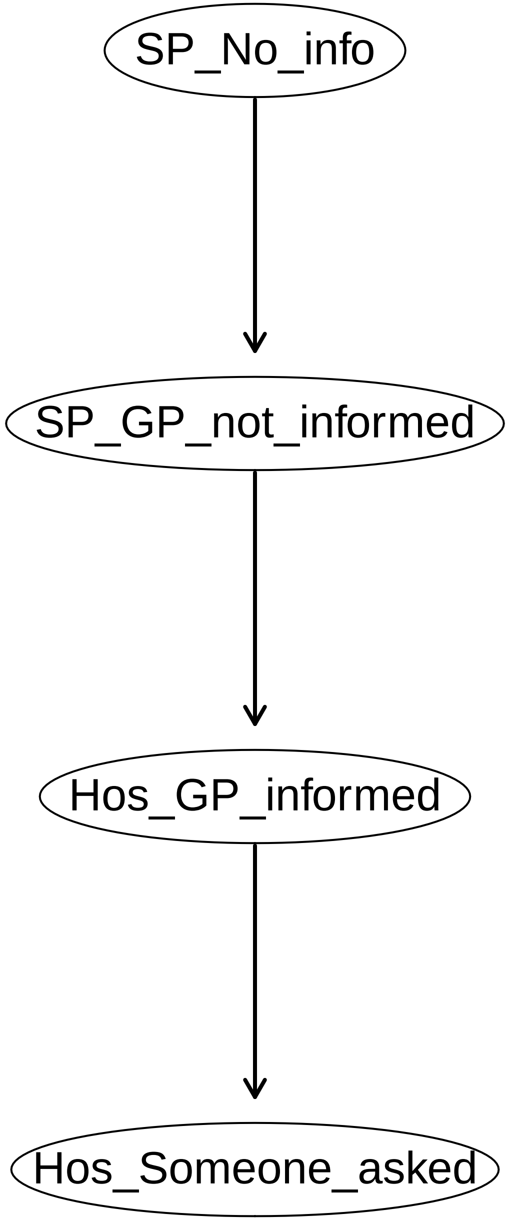
SP_No_info
SP_GP_not_informed
Hos_GP_informed
Hos_Someone_asked

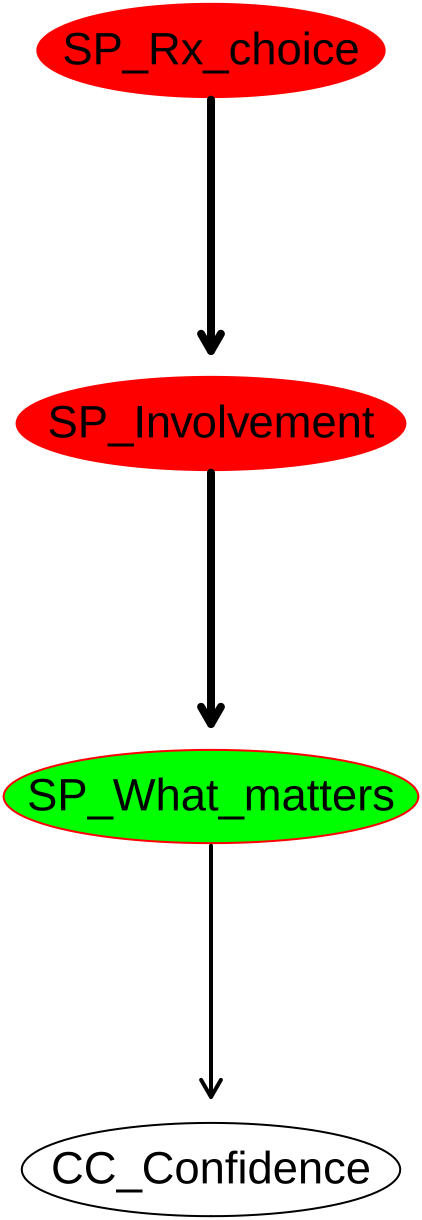
SP_Rx_choice
SP_Involvement
SP_What_matters
CC_Confidence

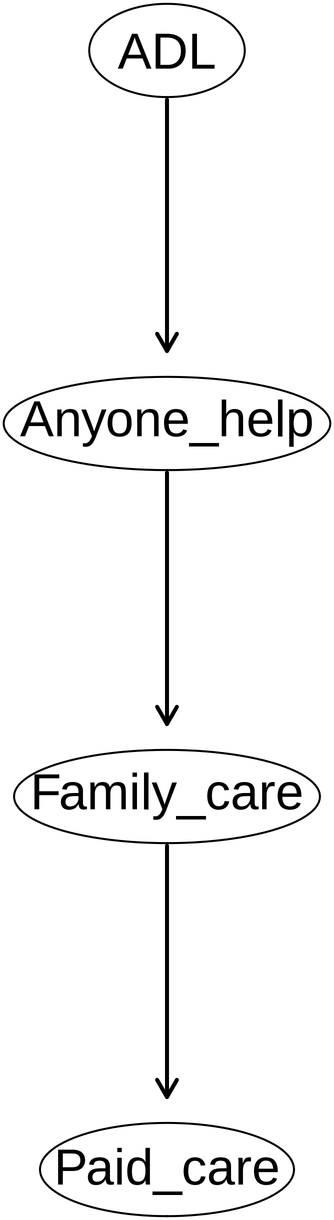
ADL
Anyone_help
Family_care
Paid_care

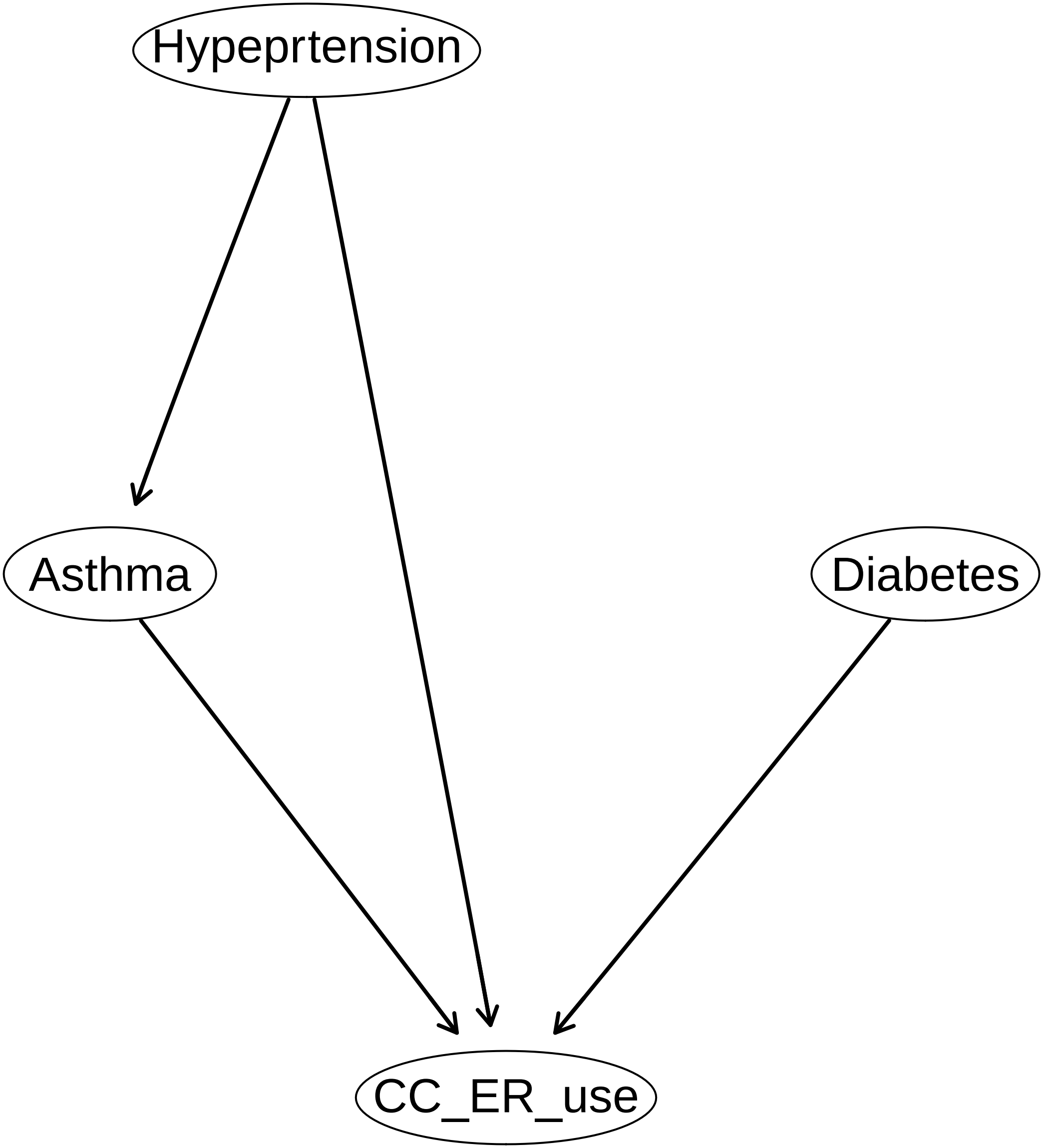
Hypeprtension
Asthma
Diabetes
CC_ER_use

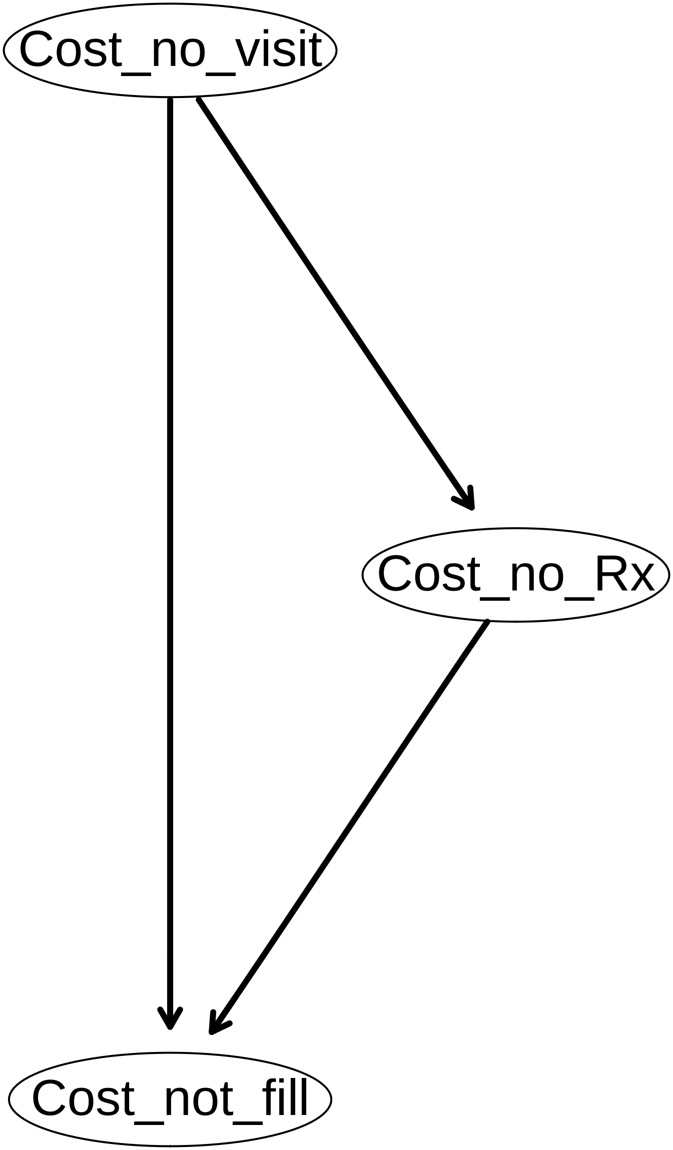
Cost_no_visit
Cost_no_Rx
Cost_not_fill

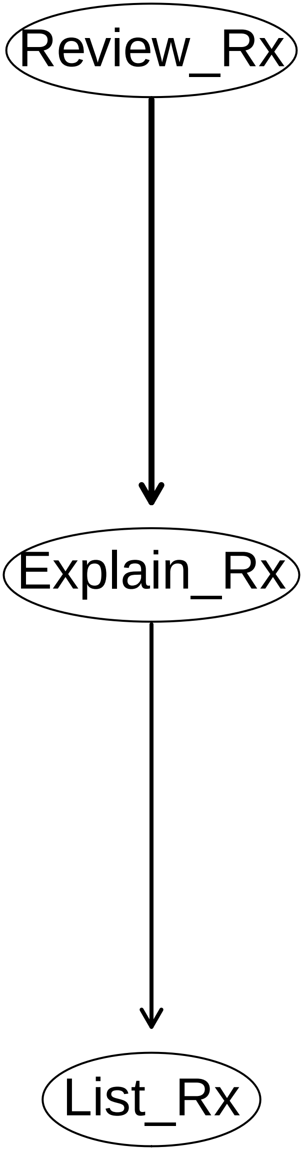
Review_Rx
Explain_Rx
List_Rx

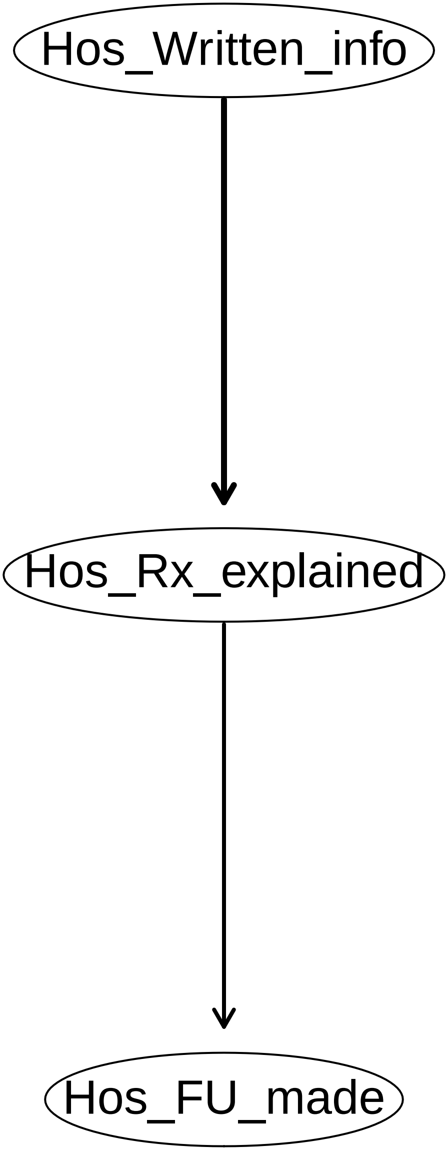
Hos_Written_info
Hos_Rx_explained
Hos_FU_made

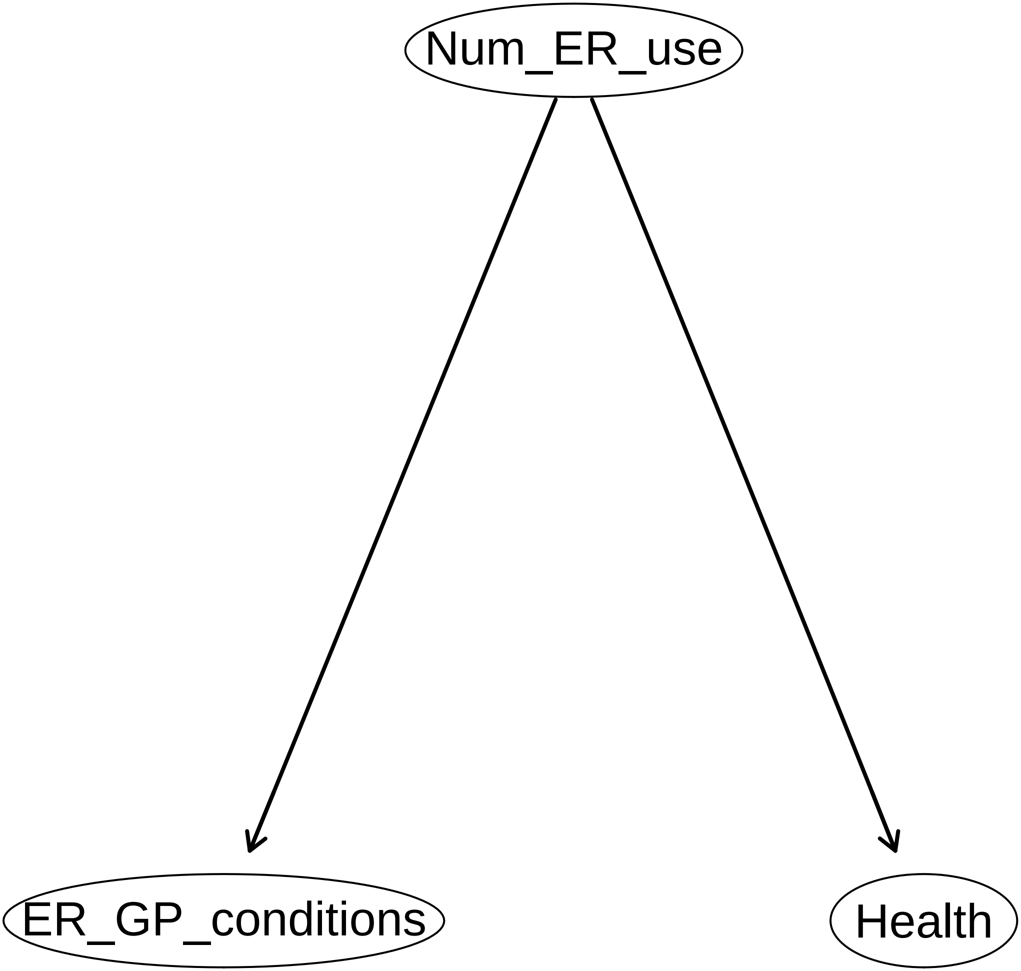

Num_ER_use
ER_GP_conditions
Health

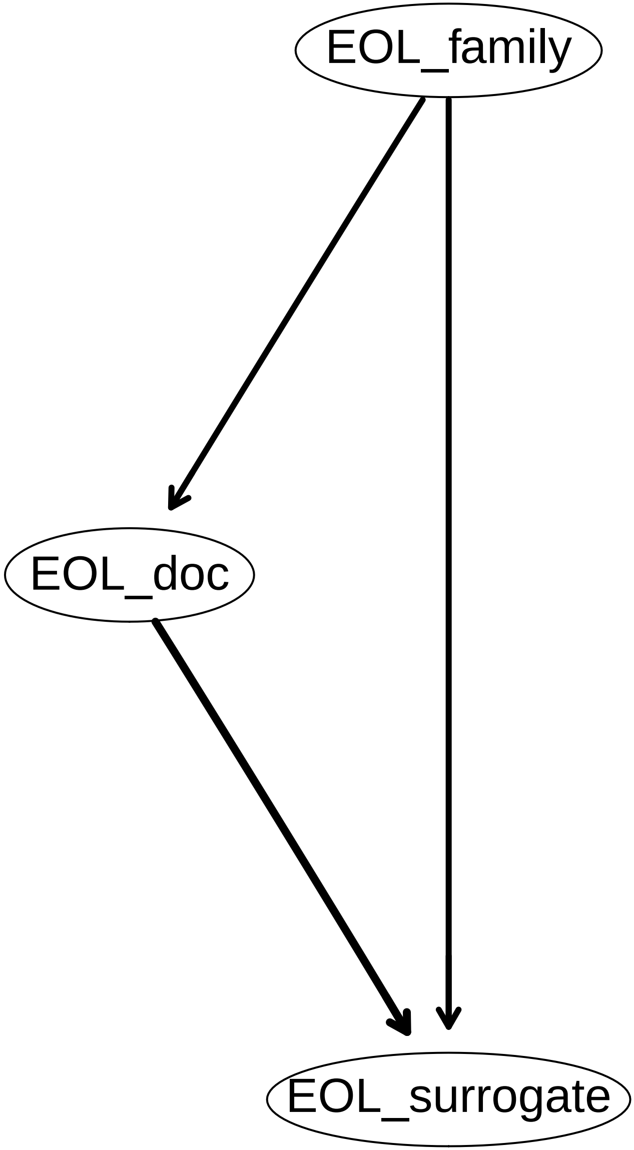
EOL_family
EOL_doc
EOL_surrogate

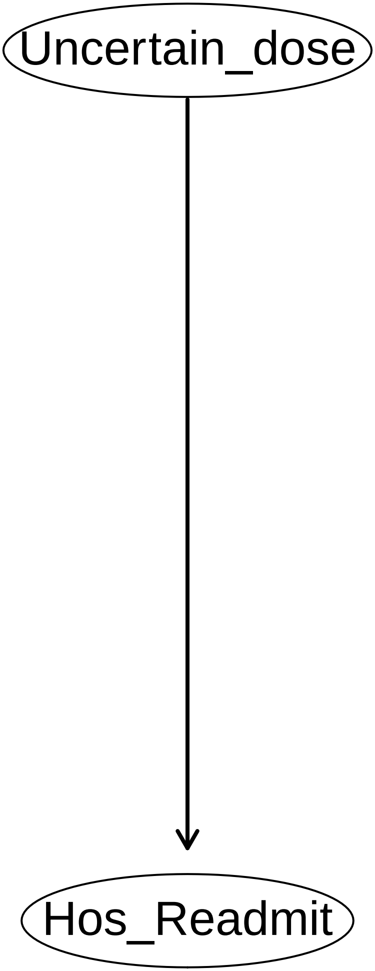
Uncertain_dose
Hos_Readmit

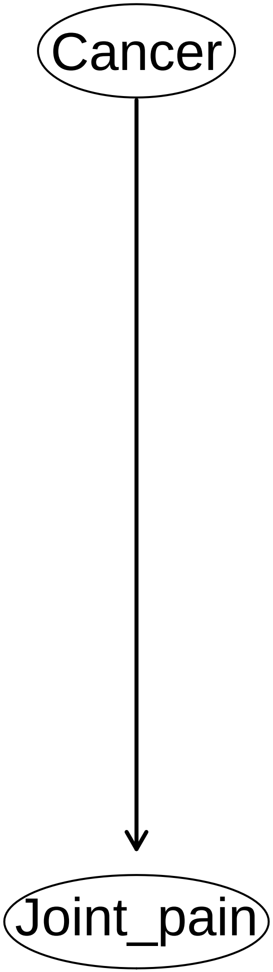
Cancer
Joint_pain

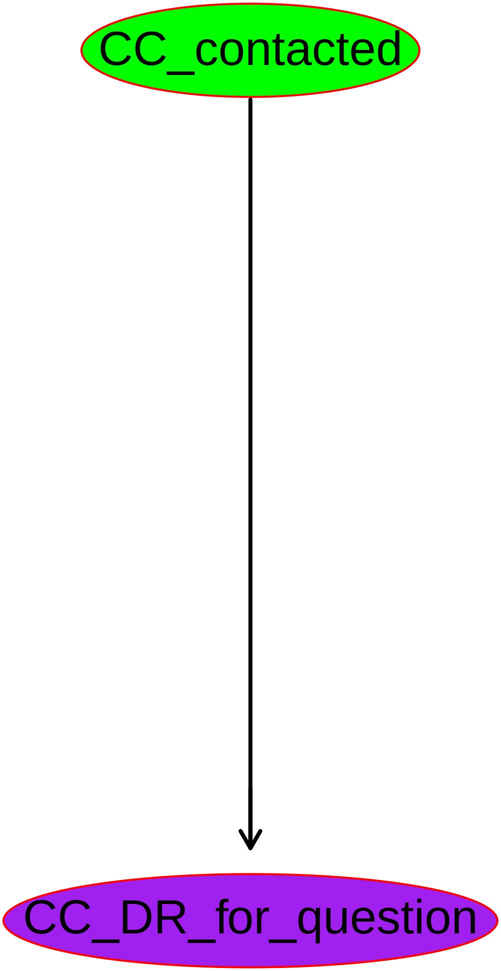
CC_contacted
CC_DR_for_question

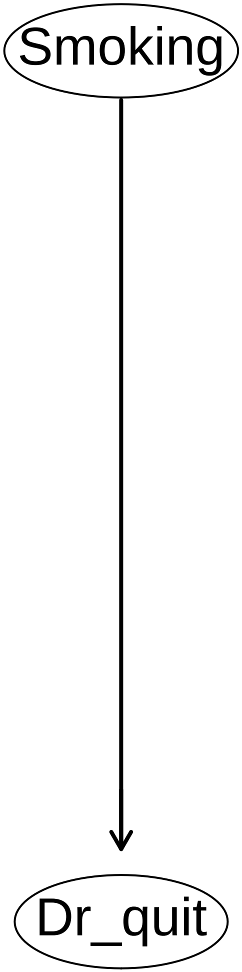
Smoking
Dr_quit

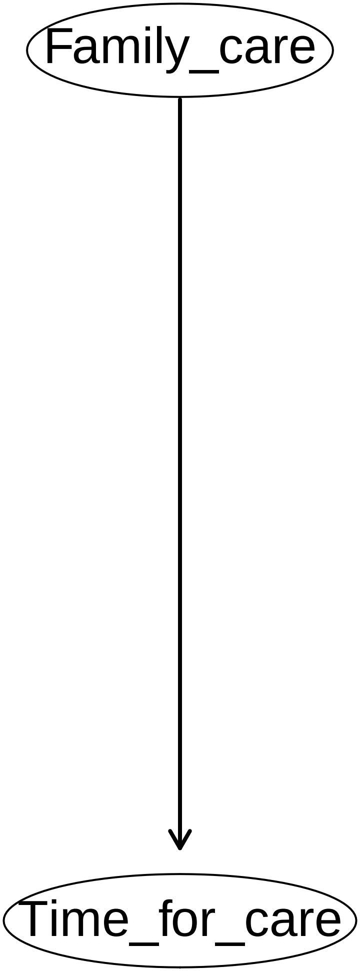
Family_care
Time_for_care
