# Supplementary material for: A network perspective of engaging patients in specialist and chronic illness care: The 2014 International Health Policy Survey
Source: PLoS One. 2018 Aug 13;13(8):e0201355. doi: 10.1371/journal.pone.0201355 (PMC6089423; doi:10.1371/journal.pone.0201355)
Supplement: S5 Appendix — (DOCX) [file pone.0201355.s005.docx]

Appendix 5. The probability distribution of the measures of patient engagement or support in chronic illness care.

(a) The probability distributions of whether patients had a treatment plan for conditions that patients could carry out in daily life.


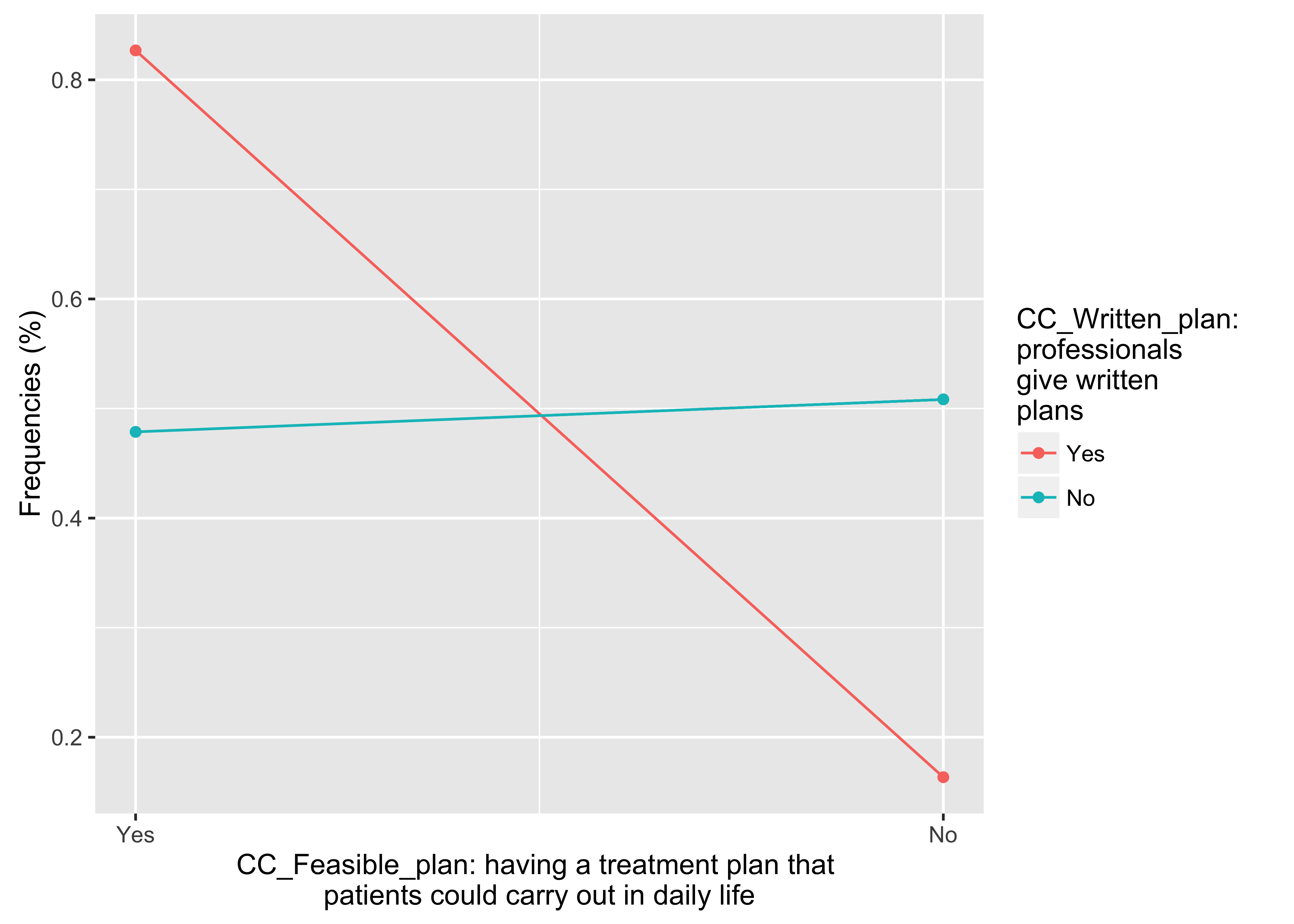


(b) The probability distributions of whether the treatment plans help patients control or manage health conditions.


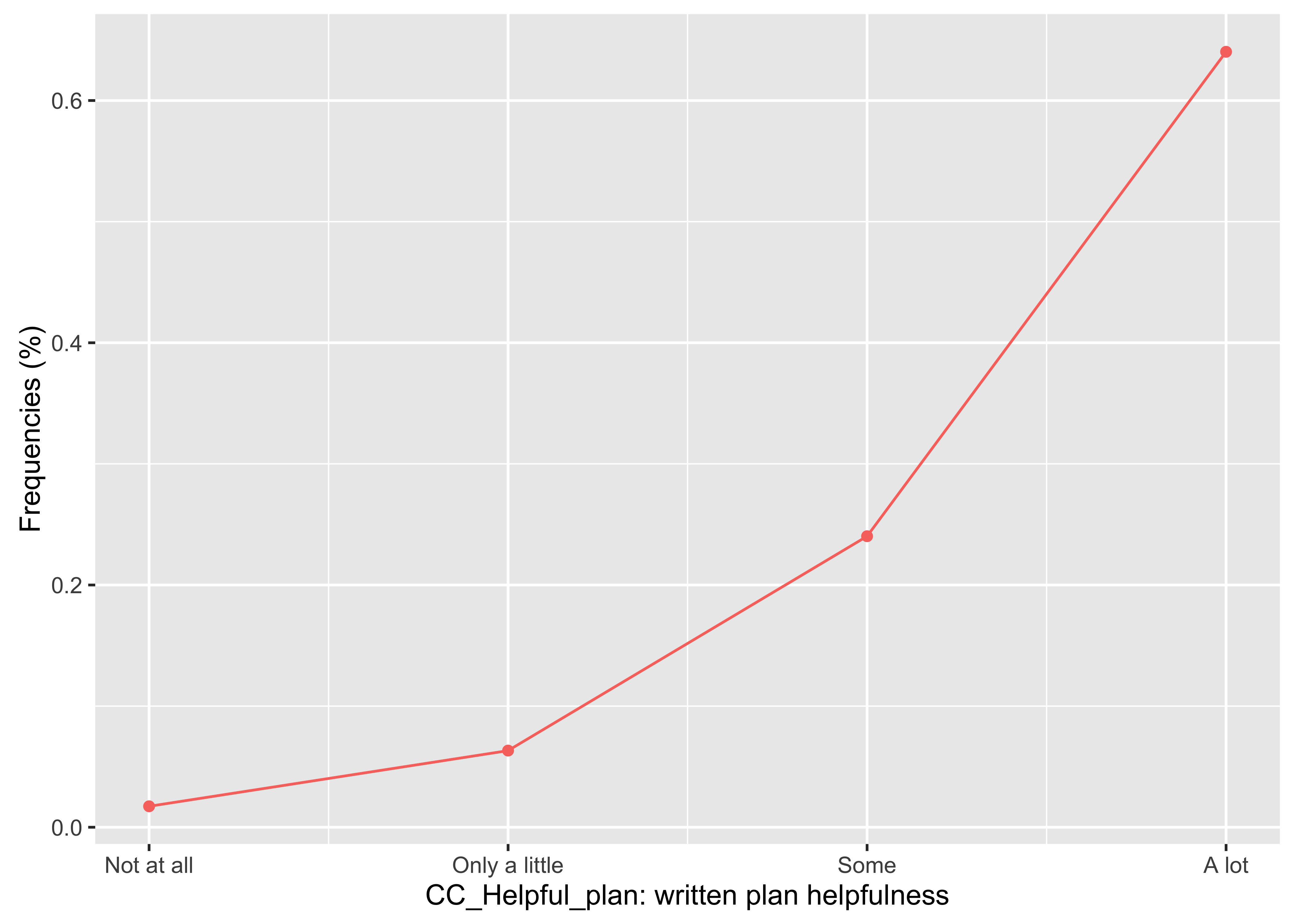


(c) The probability distribution of whether there are health professionals that patients can easily contact to ask a question or get advice for conditions between visits.


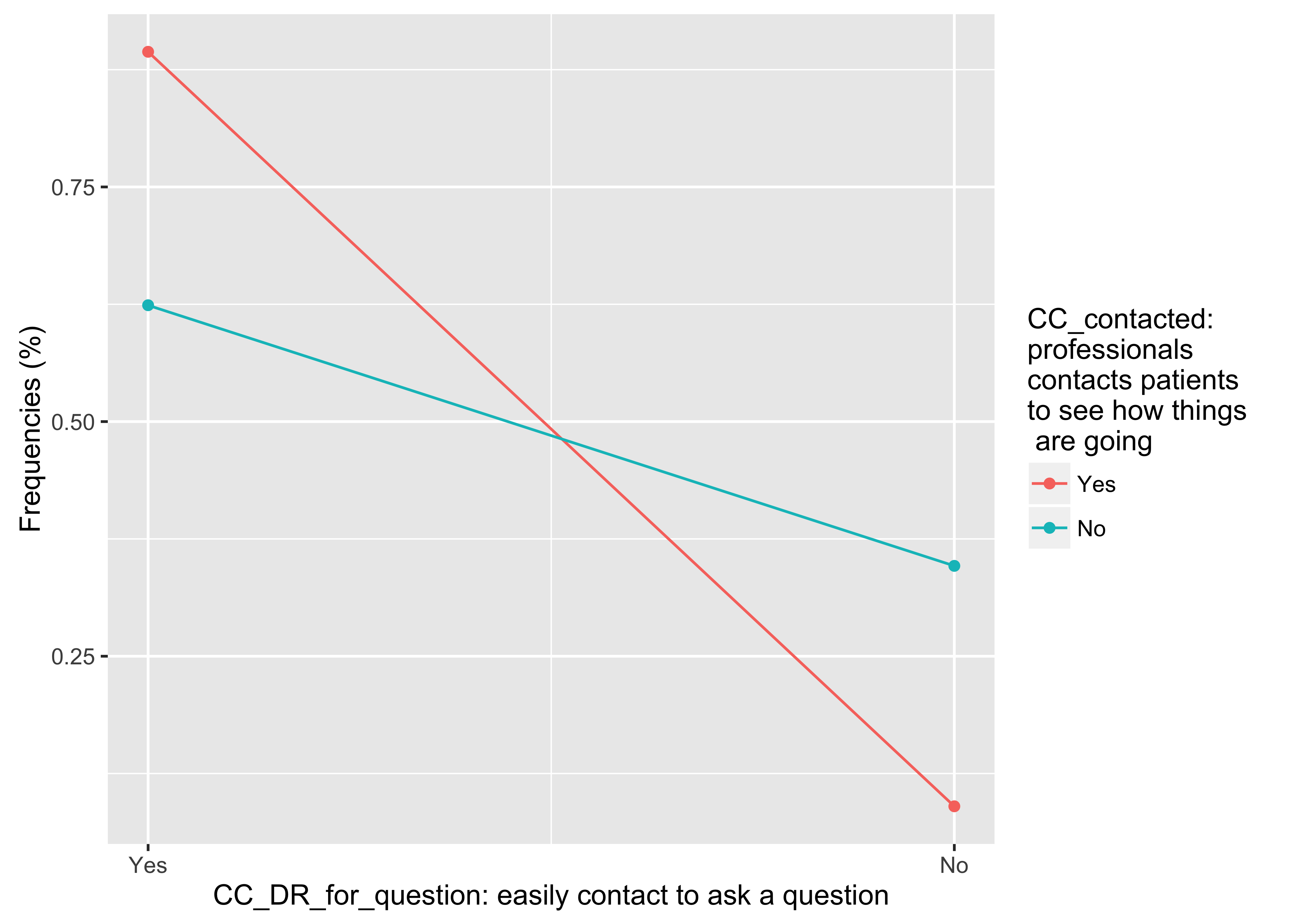


Note: The conditions included diabetes, high blood pressure, heart disease, chronic lung disease, depression, anxiety, another mental health problem, cancer, joint pain or arthritis.
