# Supplementary material for: A network perspective of engaging patients in specialist and chronic illness care: The 2014 International Health Policy Survey
Source: PLoS One. 2018 Aug 13;13(8):e0201355. doi: 10.1371/journal.pone.0201355 (PMC6089423; doi:10.1371/journal.pone.0201355)
Supplement: S6 Appendix — (DOCX) [file pone.0201355.s006.docx]

Appendix 6. The probability distributions of patient engagement or support in specialist care.

(a) The probability distribution of how often specialists involve patients as much as they want in decisions about their treatment or care.


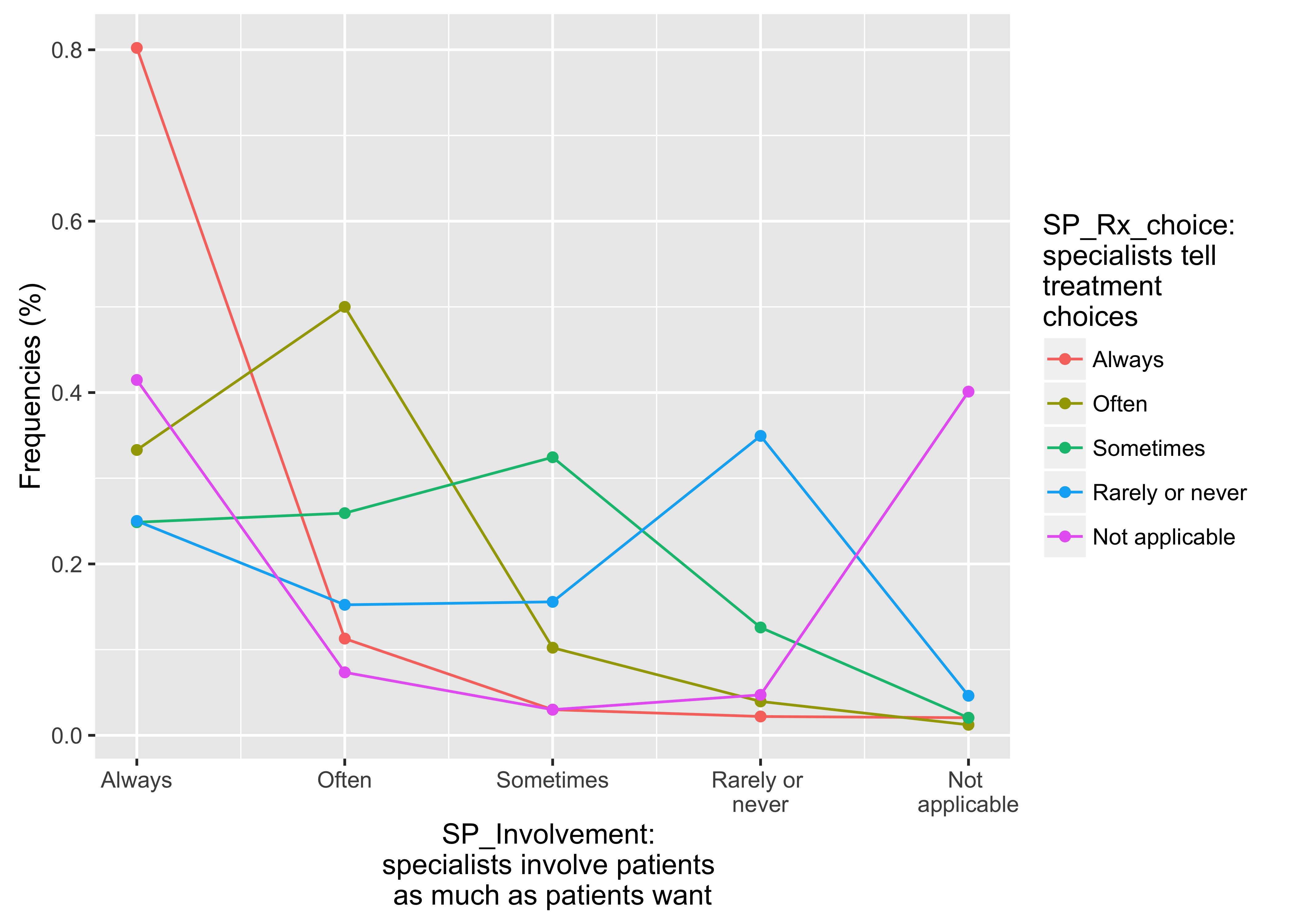


(b) The probability distribution of how often specialists involve patients as much as patients want to be in decisions about treatment or care when patients receive care or treatment from specialists.


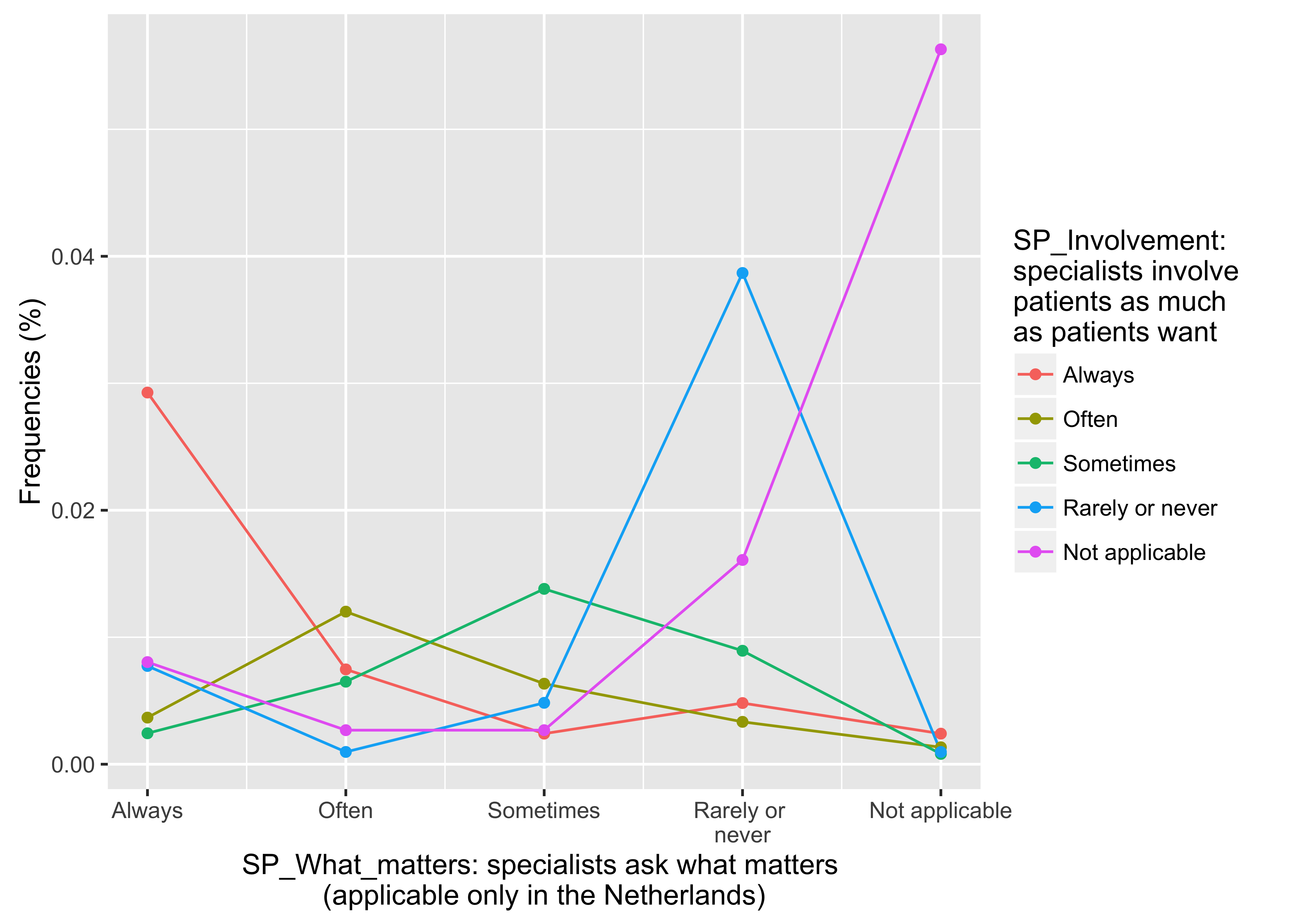


(c) The probability distribution of how patients are confident that they can control and manage their health problems.


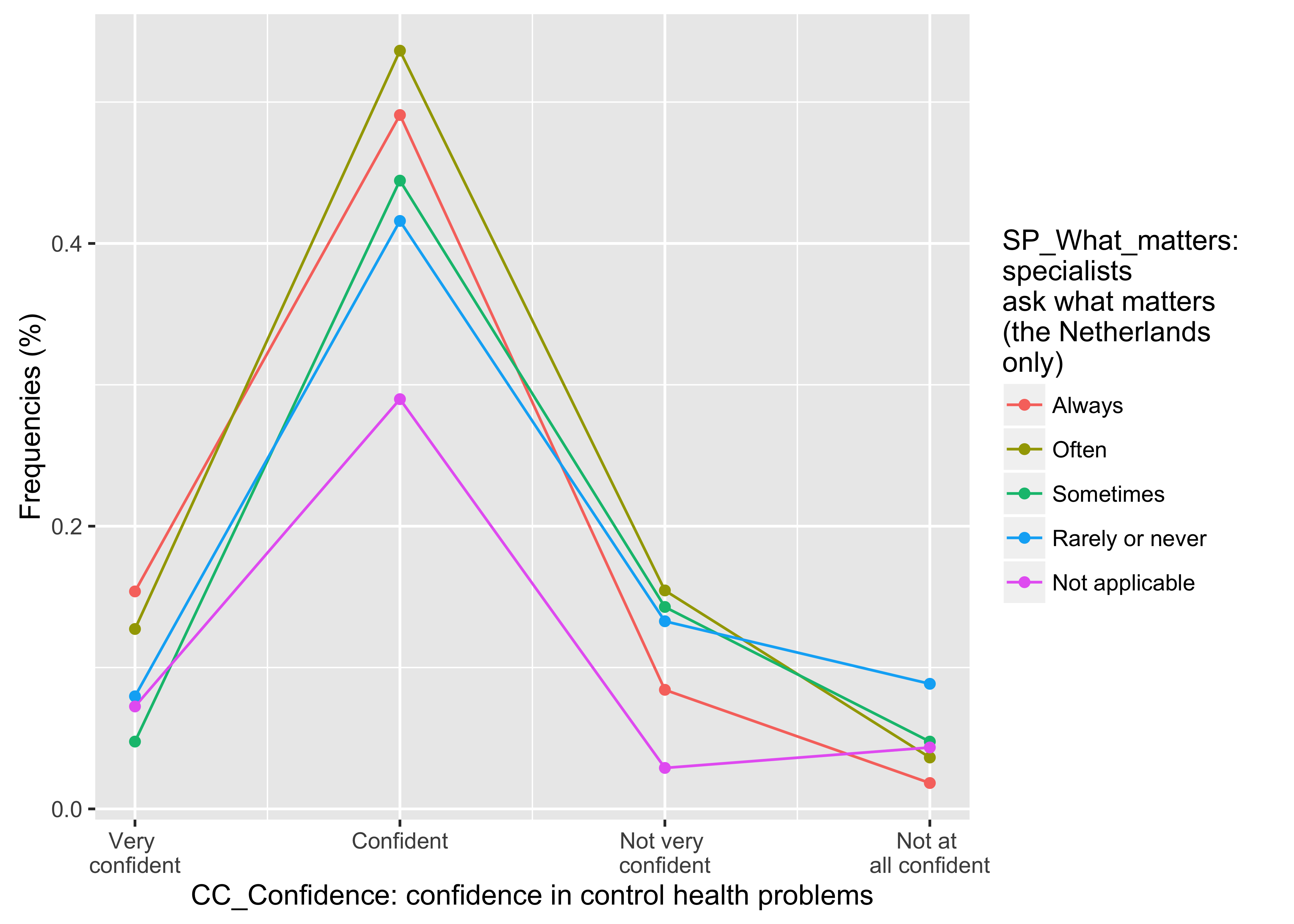


Note: Specialists are doctors that specialize in one area of health care like surgery, heart, allergy or mental health (only in Australia, Canada, France, Germany, New Zealand, Norway, Sweden, Switzerland, the United Kingdom, and the United States) or neurology (only in the Netherlands).
